# Effects of Nightshift Work on Blood Metabolites in Female Nurses and Paramedic Staff: A Cross-sectional Study

**DOI:** 10.1093/annweh/wxad018

**Published:** 2023-04-26

**Authors:** Daniella van de Langenberg, Martijn E T Dollé, Linda W M van Kerkhof, Roel C H Vermeulen, Jelle J Vlaanderen

**Affiliations:** IRAS, Institute for Risk Assessment Sciences, Utrecht University, Yalelaan 2, 3584 CM, Utrecht, the Netherlands; RIVM, Rijksinstituut voor Volksgezondheid en Milieu (National Institute for Public Health and the Environment), Antonie van Leeuwenhoeklaan 9, 3721 MA, Bilthoven, the Netherlands; RIVM, Rijksinstituut voor Volksgezondheid en Milieu (National Institute for Public Health and the Environment), Antonie van Leeuwenhoeklaan 9, 3721 MA, Bilthoven, the Netherlands; RIVM, Rijksinstituut voor Volksgezondheid en Milieu (National Institute for Public Health and the Environment), Antonie van Leeuwenhoeklaan 9, 3721 MA, Bilthoven, the Netherlands; IRAS, Institute for Risk Assessment Sciences, Utrecht University, Yalelaan 2, 3584 CM, Utrecht, the Netherlands; IRAS, Institute for Risk Assessment Sciences, Utrecht University, Yalelaan 2, 3584 CM, Utrecht, the Netherlands

**Keywords:** blood metabolites, cardio-metabolic disorders, chronobiology, circadian rhythm, fatty acids, night work, occupational health, shift work

## Abstract

Nightshift work disturbs the circadian rhythm, which might contribute to the development of cardio-metabolic disorders. In this cross-sectional study, we aimed to gain insight into perturbations of disease relevant metabolic pathways due to nightshift work. We characterized the metabolic profiles of 237 female nurses and paramedic staff participating in the Klokwerk study using the Nightingale Health platform. We performed analyses on plasma levels of 225 metabolites, including cholesterol, triglycerides, fatty acids, and amino acids. Using both principal component- and univariate-regression, we compared metabolic profiles of nightshift workers to metabolic profiles from workers that did not work night shifts (defined as day workers). We also assessed whether differential effects were observed between recently started versus more experienced workers. Within the group of nightshift workers, we compared metabolic profiles measured right after a nightshift with metabolic profiles measured on a day when no nightshift work was conducted. We observed evidence for an impact of nightshift work on the presence of unfavorable fatty acid profiles in blood. Amongst the fatty acids, effects were most prominent for PUFA/FA ratios (consistently decreased) and SFA/FA ratios (consistently elevated). This pattern of less favorable fatty acid profiles was also observed in samples collected directly after a night shift. Amino acid levels (histidine, glutamine, isoleucine, and leucine) and lipoproteins (especially HDL-cholesterol, VLDL-cholesterol, and triglycerides) were elevated when comparing nightshift workers with day workers. Amino acid levels were decreased in the samples that were collected directly after working a nightshift (compared to levels in samples that were collected during a non-nightshift period). The observed effects were generally more pronounced in samples collected directly after the nightshift and among recently started compared to more experienced nightshift workers. Our finding of a suggested impact of shift work on impaired lipid metabolism is in line with evidence that links disruption of circadian rhythmicity to obesity and metabolic disorders.

**What’s Important About This Paper?**

This study is the first to apply the Nightingale metabolomics platform to gain insight into the impact of night-shift work on perturbations in blood metabolites, relative to non-night-shift work. Further, the study, untangled night-shift work related effects on blood metabolites, demonstrating impaired lipid metabolism among night shift workers. These results support existing evidence that disruption of circadian rhythmicity by night-shift work is a risk factor for obesity and metabolic disorders.

## Background

Working in shifts concerns about 20% of the working population in Europe (Parent-Thirion *et al.*, 2018). Shift work increases the risk of a chronically disturbed circadian rhythm, meaning a misalignment of circadian rhythms of body functions, and the environmental light/dark cycle (Stevens *et al.*, 2011; Erren and Reiter, 2013). A disturbed circadian rhythm contributes to disturbed sleep/wake cycles, lifestyle-related factors (van de Langenberg *et al.*, 2018), and is associated with a range of chronic diseases including cancer (Megdal *et al.*, 2005; IARC Working Group, 2007) and cardio-metabolic disorders (Proper *et al.*, 2016). Metabolic disorders have shared underlying physiological and biochemical abnormalities, including overweight, hypertension, and glucose and lipid metabolism (Cleeman, 2001; Grundy *et al.*, 2005). Several reviews have been performed to research the impact of shift work on these intermediate outcomes that collectively increase the risk of cardio-vascular disease (CVD) and Type 2 diabetes. Strong evidence was observed for increased BMI and risk for overweight and impaired glucose tolerance (Antunes *et al.*, 2010; van Drongelen *et al.*, 2011; Proper *et al.*, 2016), yet there is insufficient support for the association between nightshift work and changes in blood lipids (Esquirol *et al.*, 2011; Proper *et al.*, 2016). Considering the chronicity of cardio-metabolic diseases and the high prevalence of shift work and related circadian disruption, a causal relation between circadian disruption and metabolic disorders would be of great public concern. Even more so since circadian disruption is also becoming increasingly prevalent in the general population due to expanding human activities over the 24 h day (Caliandro *et al.*, 2021).

In this study, we examined the impact of nightshift work on metabolic profiles in blood, in a population of 237 female nurses and paramedic staff. Metabolic profiles are assessed using the Nightingale platform, which has been successfully applied in large-scale studies to research metabolic risk factors (Soininen *et al.*, 2009; Mäntyselkä *et al.*, 2014; Würtz *et al.*, 2017; Vojinovic *et al.*, 2021). Our study includes detailed characterization of a range of aspects that might be impacted by nightshift work, including dietary aspects such as timing of eating. We compared metabolic profiles between nightshift workers and day workers using blood samples collected on days when no night work was done. In addition, among nightshift workers, assessed the acute impact of a night shift on changes in metabolic profiles using blood samples that were collected on the morning directly after a nightshift.

## Methods

### Study set-up and sample collection

The study design of the Klokwerk study is described elsewhere (van de Langenberg et al., 2019, 2018). In brief, the study population consisted of 237 female nurses and paramedic staff between ages 18 and 65. To research effects of nightshift work on metabolic biomarkers we compared nightshift workers with participants who did not perform nightshift work in the last 5 years, defined as ‘day-workers’ (control subjects). Night-shift workers worked in a rotating shift-work schedule and organize their own schedules (self-schedule). We collected (non-fasting) blood samples during time-periods in which study participants did not conduct nightshift work (defined as a day session). During these day sessions, samples were ideally collected on days and times when participants were least disrupted by their rotating-shift working schedule: while working afternoon shifts (i.e. their working schedule did not affect their preferred time of waking up) and as long as possible after their last night shift (median of 9 days). A subset of the participants (*n* = 90) has been studied more extensively, with a more demanding study protocol. Within this subset, we aimed to collect blood samples twice per participant during day sessions (during two separate sessions). To analyse acute effects of nightshift work, within this subset, we collected one (non-fasting) blood sample in the morning immediately after a nightshift session for nightshift workers (*n* = 69), and compared with samples taken during day work, at the start of the shift. Biological sampling was conducted at the end of at least two consecutive day- or nightshifts. ‘Day work’ was defined as all work that does not cover the definition of a ‘night shift’. Besides traditional working hours during the day, day work also includes morning and afternoon shifts. We defined nightshift work as working at least one night shift every 6 weeks in a rotating schedule. We defined a night shift as having worked at least one hour between midnight and 06:00 AM. We performed additional analyses, for which nightshift workers were divided into experienced nightshift workers (started working night shifts over 5 years ago), and recently-started nightshift workers (less than 2 years ago).

We approached potentially eligible participants via a screening questionnaire that was distributed among nurses in five selected hospitals in the Netherlands. All participants signed an informed consent. Inclusion of the participants took place between February 2015 and February 2017. To participate in the study, subjects had to agree to blood sampling, and fill out the questionnaires. We excluded current smokers and former smokers who quit smoking <6 months before study inclusion [<100 cigarettes during lifetime were considered nonsmokers (Centers for Disease Control and Prevention (CDC) 2011)]. We excluded participants that were pregnant (or had been in the six months before inclusion); were undergoing fertility treatment; had ever been diagnosed with cancer (excluding nonmelanoma skin cancer), high blood pressure (using beta-blockers), or diagnosed cardiovascular disease; and used melatonin supplementation or medication for chronic disorders including diabetes. We approached 257 participants, 18 were excluded based on the screening questionnaire. Two participants were excluded due to missing blood samples. The study was approved by the Institutional Review Board of the University Medical Centre Utrecht, the Netherlands (14-611D, NL51501.041.14).

### Covariates

We acquired participant characteristics including age, BMI (body mass index, dividing weight in kilograms by height in meters squared), education, marital status, and total duration of nightshift work in years. Chronotype was self-assessed by the participants using an item of the Horne-Ostberg scale (Horne and Ostberg, 1976) on diurnal preference with five categories (obvious morning preference, more morning than evening preference, more evening than morning preference, obvious evening preference, no specific type). We used a questionnaire also designed to establish the chronotype in shift workers [the Horne-Östberg Morningness-Eveningness Questionnaire (MEQ)] (Horne and Ostberg, 1976). MEQ determines chronotype using questions on diurnal preferences. We used one item from the MEQ, a self-assessment of the chronotype of the participants by choosing from different categories. This self-assessment of one’s chronotype gave a similar result (R-square of 0.8) to the 19-item questionnaire in a validation study by Roenneberg *et al*. (Zavada *et al.*, 2005; Roenneberg *et al.*, 2007). We collected time and date for each blood sample. We assumed a 24 h cyclical trend and assess an effect of ‘time of blood sampling’ by performing cosinor analyses using the cosionor2 package in R (Cornelissen, 2014).

More detailed information on confounding factors was available for a subset of the participants (*n* = 90, of which 69 are nightshift workers). We asked participants to specify timing of eating and type of nutrition (product and quantity). The nutritional intake has been performed by a trained nutritionist. Using these 24 h dietary logs we calculated timing since last meal before blood draw. We applied a Dutch nutrient database (NEVO, 2016) to calculate total energy intake and macronutrients, including saturated fat intake. In a previous study (van de Langenberg *et al.*, 2018) we have addressed key exposures associated with nightshift for this subset. Reasons for the variability in the response to nightshift work include meal timing and intake of (saturated) fat of the participants, which we included in our analyses in the present study.

### Metabolic profiling and limit of detection

The plasma samples were analysed using Nightingale Health’s blood biomarker analysis platform, a high-throughput NMR spectroscopy technique which allows for a comprehensive profiling of circulating lipids and metabolites (Soininen *et al.*, 2009), in August 2017. The metabolites collectively represent a molecular signature of systemic metabolism (Soininen et al., 2015, 2009), including quantification of several sizes of low-density intermediate-density and high-density lipoprotein lipids and subclasses, their contents (cholesterol, triglycerides, phospholipids), fatty acids, fatty acid compositions, and ratios of the aforementioned metabolites in relation to total concentration of their respective subclass (omega-3 fatty acid to total fatty acid ratio), ketone bodies, amino acids, as well as glycolysis and gluconeogenesis-related metabolites (like glucose, citrate) in absolute concentration units. Overall, the metabolite levels observed in our population broadly fall within the distributions commonly observed in general population cohorts and the mean success rate was 99.02% (above the limit of detection, results not shown) (31). Lowest mean success rate was observed for XXL particle size of very-low density lipoproteins (VLDL), XL.VLDL and L.VLDL. 12% for XXL, 15.8% for XL, and 15% for L particles were below the detection limit, defined by Nightingale as ‘very low concentration’ (Do *et al.*, 2018; Emwas *et al.*, 2019). This is commonly observed, in particular in samples from generally healthy individuals, since the concentration levels of large VLDL lipids are biologically known to be very close to zero.

### Data quality and preparation

Metabolite measurements below the limit of detection were imputed with an R-script that employed Maximum Likelihood Estimation to impute values under the limit of detection (LOD) (Lubin *et al.*, 2004). Less than 10% of the covariate-values were missing, which we imputed using the Predictive Mean Matching function (‘pmm-method’) of the ‘multivariate imputation by chained equations’ (MICE) package in R (Buuren and Groothuis-Oudshoorn, 2011). The data is analysed using R (R version 3.6.3).

### Data analysis

#### Principal component regression

We used default R function ‘*prcomp*’ to conduct principal component analyses (PCA) within three sets of (highly correlated) groups of metabolites: ‘cholesterol, glycerides and lipoproteins’ (*n* = 28 compounds); ‘fatty acids’ (*n* = 16 compounds); and ‘amino acids’ (*n* = 14 compounds). In addition, we also applied PCA on all measured metabolites (*n* = 225) to identify potential underlying patterns across metabolite groups (Ivosev *et al.*, 2008). Principal components that explained over 70% of the variance within for each metabolite group were included as outcome into a linear mixed-effects regression model (to accommodate the repeated measures design) (Cnaan, Laird and Slasor, 2005); [LMER package for R (Bates *et al.*, 2015)]. All regression models are adjusted for age at time of blood-draw (model A). In a second confounder model, analyses were also adjusted for BMI (model B). In a third model, we further adjusted analyses for chronotype, and blood-sampling time (model C). In the main analysis, we combined recent and experienced nightshift workers into one category. Sensitivity analyses were conducted to assess whether effects differed between recently started (<2 years) and experienced (>5 years) nightshift workers. The same approach was applied in the analyses that aimed to assess the long-term impact of nightshift work on metabolic profiles measured during a non-nightshift day, as well as in the analyses that aimed to assess the short-term impact on metabolic profiles measured in blood samples that were collected on the morning directly after a nightshift.

#### Univariate analyses

In addition to the principal component regression we also performed univariate mixed-effect linear regression models on all 225 individual markers independently. We graphically present a subset of 58 a-priori selected metabolites that are related to cardio-metabolic disease endpoints in previous research (Sappati Biyyani *et al.*, 2010; Auro *et al.*, 2014; Camacho *et al.*, 2019), using a forest plot. In this plot, each metabolic measure is expressed as a difference in means in SD units.

## Results

### Description of the study population

The study population (described in Table 1) consisted of 237 participants, of which 94 were day-workers, and 143 were nightshift workers. For a proportion of the study population (*n* = 147, of which 74 where nightshift workers), only one sample collected during a day session was available, and no dietary information was available. The participants were between the 19 and 65 years old, with a median age of 42.5. 34.0% of the participants were considered overweight (BMI over or equal to 25). A higher percentage of the nightshift workers considered themselves a clear evening type (15.4%), compared to day workers (7.7%). Table 1 in the supplementary material (S1 Appendix) is an extended version of Table 1, in which we further stratified the nightshift worker group into recent nightshift workers, and experienced nightshift workers.

**Table 1.** General characteristics of study population (n = 237, total number of observations = 361).^a^

Number of samples/observations	Day workers (*n* = 94)	Nightshift workers (*n* = 143)
	114 Day-shift samples	181 Day-shift samples, 66 nightshift samples
Age in years (mean ± SD)	44 ± 13	39 ± 12
Body mass index (mean ± SD)^b^	24.9 ± 4.2	26.6 ± 5.2
Years of nightshift work experience (mean ± SD)	–	14.5 ± 11.4
*Chronotype*		
Clearly morning person	24.1%	10.3%
More morning than evening person	34.1%	26.5%
No preference	14.3%	19.9%
More evening than morning person	19.8%	27.9%
Clearly evening person	7.7%	15.4%

^a^Missing data excluded from frequency table.

^b^Weight in kilograms divided by height in meters squared.

### Nightshift workers compared to day workers

#### Principal component regression

**Figure 1.**
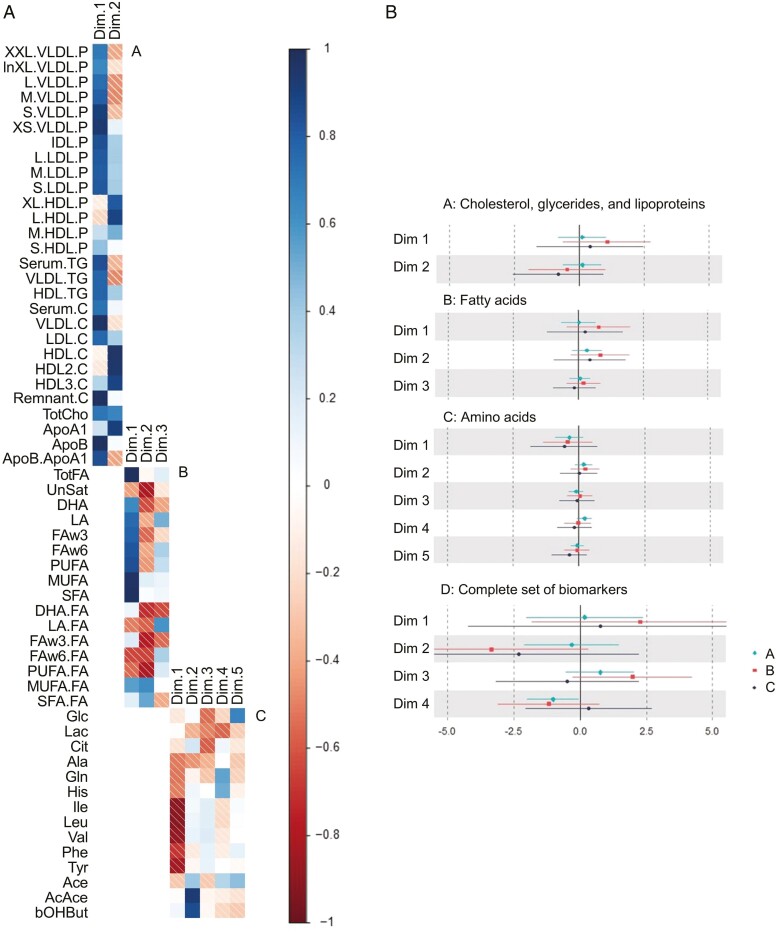
PCA loading plots and associations of linear regression analyses (confidence intervals) between nightshift work and components. a: Principal component loading plots: fully-shaded squares indicate positive loading, whereas striped squares indicate negative loadings. b: Associations for nightshift work and dimensions (components). Non-fasted blood samples of nightshift workers (both recent and experienced, *n* = 143) were compared to those of day workers (*n* = 94). A = minimal confounder model, associations are adjusted for age. B = confounder model, associations are adjusted for age, and BMI. C = full-covariate model, associations are adjusted for age, BMI, chronotype, and blood sampling time.

Results from the principal component regression are presented in Fig. 1 and described below per compound group and for the complete set of biomarkers. We describe the loadings for the first two components per compound group, while loadings for the other components are described in S2 Appendix (Fig. 1). S2 Appendix also reflects the *scree* plots of the eigenvalues, demonstrating the variance collectively explained by the components.

#### Cholesterol, glycerides, and lipoproteins

Two principal components were needed to capture 70% of the variation in this compound group. Component 1 is characterized by positive loadings for all cholesterol lipoproteins, except for the large and extra-large particle sizes of high-density lipoprotein (HDL) levels, Fig. 1. Component 2 is characterized by negative loadings for very low-density lipoprotein (VLDL) and triglyceride, and strong positive loadings of HDL cholesterol and apolipoprotein A-I. Within the full-covariate model (model C, Fig. 1), we observed a positive association between nightshift work and component 1 (β 0.41, BI −1.65, 2.47) and a negative association with component 2 (β −0.81, BI −2.55, 0.93). These associations were absent or less pronounced in models A and B.

#### Fatty acids

Three principal components were needed to capture 70% of the variation in this compound group. Component 1 is characterized by high positive loadings for total fatty acids (TotFA), saturated fatty acids (SFAs), and monounsaturated fatty acids (MUFAs), and negative loadings for unsaturated fatty acids; especially ratios of polyunsaturated fatty acids to total fatty acids (PUFA/FA) and ratios of omega-6 fatty acids to total fatty acids (FAw6/FA). Component 2 is characterized by negative loadings for the unsaturated fatty acids (all except the MUFAs), and positively driven by SFA/FA ratios. We did not observe evidence for an association between nightshift work and component 1 in models A, B, or C. We did observe a positive association with component 2 in all three models (β 0.42, BI −1.03, 1.87, in model C).

#### Amino acids

Five principal components were needed to capture 70% of the variation in this compound group. Component 1 is characterized by negative loadings for all amino acids; in particular isoleucine, leucine, valine, and tyrosine and to a lesser degree alanine, glutamine, and histidine. Component 2 was primarily characterized by positive loadings for ketone bodies (acetoacetate and β-hydroxybutyrate). Component 1 was negatively associated with nightshift work in models A, B, and C (β −0.55, BI −1.82, 0.72, in model C). We did not observe evidence for an association between nightshift work and components 3–5 in models A, B, or C.

#### Complete set of biomarkers

Four principal components were needed to capture 70% of the variation in the complete set of biomarkers. Component 1 is characterized by positive loadings for most of the lipoproteins. Component 2 is characterized by negative VLDL loadings and positive HDL, LDL, and PUFA loadings. S2 Appendix reflects the principal component (PC) loading plots for the complete set of components. Component 1 was positively associated with nightshift work in models A, B, and C (β 0.77, BI −4.23, 5.76, in model C). Component 2 was negatively associated with nightshift work in models A, B, and C (β −2.32, BI −6.85, 2.21, in model C). For components 3 and 4, associations with nightshift work varied considerably across models A, B, and C.

### Univariate analyses

Within the univariate regression analyses (visually displayed in S3a Appendix), we observed negative associations between nightshift worker and levels of large particle size HDL levels and a positive association with VLDL. For all noteworthy associations at least one of the covariate models resulted in a null effect. Based on the full-covariate model (model C), most prominent associations were observed for TotFAs (elevated compared to day workers), LA (linoleic acid)/FA (decreased), FAw6/FA (decreased), PUFA/FA ratios (decreased), MUFA/FA ratios (elevated), and SFA/FA ratios (elevated); see S3a Appendix. Effects were most consistent for PUFA/FA (consistently decreased) and SFA/FA (consistently elevated), while for the other noteworthy associations at least one of the covariate models resulted in a null effect. The results for the amino acids observed in our univariate analyses (S3a Appendix) agree with the effects we observed for the PCA; glutamine, isoleucine, and leucine levels were consistently elevated when comparing nightshift workers with day workers (in all three covariate models). In addition, in our univariate models, histidine levels were positively associated with nightshift work. Similar results were observed within our subset for which we had more elaborate covariates available (S3b Appendix).

### Impact of years of nightwork experience on observed associations

Among the sensitivity analyses where we made a distinction between experienced and recently-started nightshift workers (S4 Appendix), effects were more pronounced for all components in the categories of metabolites: ‘cholesterol, glycerides and lipoproteins’ and ‘fatty acids’. Within the category of amino acids component 1 (characterized by negative loading for all amino acids; in particular isoleucine, leucine, valine, and tyrosine) was more pronounced and significantly decreased for recently-started nightshift workers (β −1.73, BI −3.42, −0.04, S4 Appendix). No statistically significant associations were observed for the association between recently starting nightshift work and component 2 of the amino acids. In the univariate analyses, we observed more pronounced decreased levels of large and extra-large particle sizes of HDL due to nightshift work for recently-started nightshift workers compared to experienced nightshift workers. Also, associations between nightshift work and levels of serum TG and VLDL.TG were more pronounced for recently started nightshift workers than for experienced nightshift workers. Decreased FAw6/FA, PUFA/FA, and LA/FA ratios and elevated MUFA/FA and SFA/FA ratios were primarily visible among recently started nightshift workers and much less so among experienced nightshift workers (S4 Appendix). The amino acids histidine, isoleucine, leucine, valine, and phenylalanine were significantly elevated when comparing recently-started nightshift workers with day workers, while for experienced nightshift workers these effects were diluted. For recently-started nightshift workers alanine and glucose levels were non-significantly elevated whereas for β-hydroxybutyrate decreased levels were observed; for these markers an opposite effect was observed for experienced nightshift workers. For the other markers the associations did not differ majorly between recently started and experienced nightshift workers (S4 Appendix).

### Acute effects measured directly after a nightshift

For a subset of the nightshift workers (*n* = 69), a blood sample collected the morning immediately after a nightshift was compared with samples taken during day work (reference group). The first two components are described in the main text, the other components are described in S5 Appendix, unless they are considered relevant for the interpretation of the results. S5 Appendix also reflects the *scree* plots of the eigenvalues, demonstrating the variance collectively explained by the components.

### Principal component analyses

#### Cholesterol, glycerides, and lipoproteins

**Figure 2.**
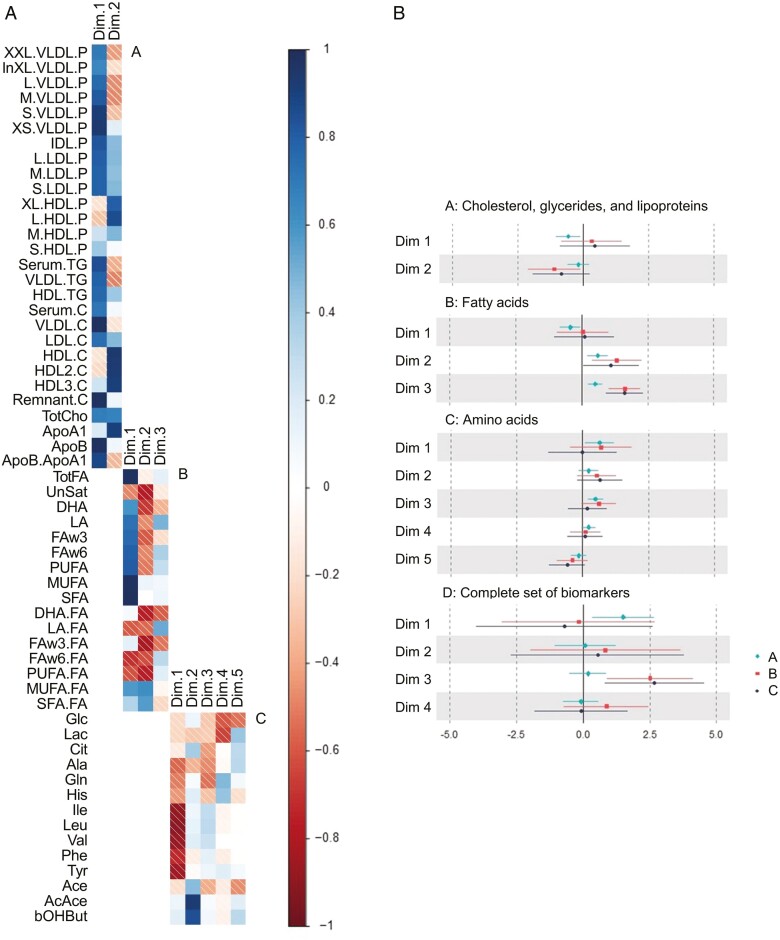
PCA loading plots and associations of linear regression analyses (confidence intervals) between acute nightshift work and components (dimensions). a: Principal component loading plots: fully-shaded indicate positive loading, whereas stripped squares indicate negative loadings. b: Acute effects of the nightshift directly before blood sampling compared with blood sampling during a day session among nightshift workers (*n* = 69, 184 samples). A = minimal confounder model, associations are adjusted for age. B = confounder model, associations are adjusted for age, BMI, and blood sampling time. C = full-covariate model, associations are adjusted for age, BMI, chronotype, timing of last meal, and blood sampling time.

Component 1 is characterized by positive loadings for all cholesterol lipoproteins, except for the large and extra-large particle sizes of HDL. No clear association was observed for component 1 within the category ‘cholesterol, glycerides, and lipoproteins’, Fig. 2. Component 2 (characterized by negative loadings for VLDL cholesterol and triglyceride, and positive loadings of HDL cholesterol and apolipoprotein A–I) was consistently decreased for all covariate models after working a nightshift before blood sampling (β 0.92, BI −0.16, 2.01 for the full-covariate model, Fig. 2). These results were similar to what was observed in our analysis comparing nightshift workers with day workers.

#### Fatty acids

Component 1 is characterized by high positive loadings for TotFA, SFAs, and MUFAs, and negative loadings for UFAs; especially ratios of PUFA/FA and FAw6/FA. No clear association was observed for this component (Fig. 2). We observed significant acute effects for component 2 and 3. Component 2 is characterized by negative loadings for the UFAs (all except the MUFAs), and positively driven by SFA/FA ratios and is consistently elevated directly after working a night shift, for all three covariate models (β 1.11, BI 0.05, 2.17 for the full-covariate model). Component 3 [characterized by negative loadings for docosahexaenoic acid (DHA) and DHA/FA and ratio of omega-3 fatty acids to total fatty acids (FAw3/FA), and positive loadings for LA and FAw6] was consistently elevated as well (β 1.62, BI 0.92, 2.33 for the full-covariate model, Fig. 2). In general effects in fatty acid levels were more pronounced in these samples (collected directly after a nightshift) than in samples collected during a non-nightshift period to compare nightshift workers to controls.

#### Amino acids

Component 1 within the category ‘amino acids’ (negatively loaded by all amino acids, with highest negative loadings for the amino acids: isoleucine, leucine, valine, and tyrosine) was elevated directly after working a night shift. However, in the full-covariate model, where we adjusted for meal timing, a null-effect was observed. Component 2 (primarily characterized by positive loadings for ketone bodies) was consistently elevated directly after working a night shift, for all covariate models.

#### Complete set of biomarkers

S5 Appendix reflects the principal component (PC) loading plots for the complete set of biomarkers/metabolites. No significant association was observed between regularly working night shifts and the components of the complete set of biomarkers, except for the full-covariate model of component 3, characterized by high positive HDL loadings (β 2.67, BI 0.80, 4.53 Fig. 2). Results from univariate analyses (S6 Appendix) were generally in line with the results from principal component regression described above.

## Discussion

Our main finding is an association between nightshift work and unfavorable patterns in fatty acid profiles detected in blood. We also observed associations with amino acid concentrations and lipoproteins (especially HDL-cholesterol, VLDL-cholesterol, and triglycerides). Differences in biomarker levels were more pronounced when comparing samples collected directly after a nightshift to samples collected during a non-nightshift period than when we compared samples collected from nightshift workers to controls.

Our findings of an association between nightshift work and unfavorable fatty acid profiles in blood are in line with a cross-sectional metabolic profile of shift workers and day workers, where an adverse effect of long-term circadian misalignment on lipids was suggested (Huang *et al.*, 2021). In agreeance with our study, most prominent effects were observed for unsaturated fatty acids and linoleic acid (LA) metabolism (Huang *et al.*, 2021). We observed a higher degree of saturation of fatty acids and lower PUFA/FA ratios, which have been associated with both an unhealthy diet and increased risk of cardio-metabolic diseases (Akbaraly *et al.*, 2018). Our study design allowed us to research acute effects immediately after a night shift (utilizing the comparison within the nightshift work group immediately after an exposure). The acute effects in terms of ratios and unfavorable fatty acid profiles were consistent with the effects when comparing nightshift workers with day workers. Our results expand on previous findings describing acute nightshift work could particularly affect (anti-obesogenic) polyunsaturated fatty acids (PUFAs) (Huang *et al.*, 2021). Although all PUFAs are generally more beneficial for cardio-metabolic health compared to SFA, a distinction can be made between omega-3 and omega-6. Diets deficient in omega-3 fatty acids, and with increased levels of the pro-inflammatory omega-6 fatty acids, might lead to elevated triglycerides levels (Simopoulos, 2002; Blasbalg *et al.*, 2011; Mejía-Barradas *et al.*, 2014) and reduced HDL-cholesterol levels (Poudyal *et al.*, 2011) which often accompany metabolic disorders (McCracken *et al.*, 2018). Interestingly, we observed a high omega-6/omega-3 ratio, directly after a night shift. Recently, an obesogenic diet and its main component, saturated fatty acids, has also been shown to disturb circadian secretion of metabolic hormones (Martchenko and Brubaker, 2021). Since we have previously described a change in fat quality during the nightshift in our study population (van de Langenberg *et al.*, 2018); there is an active question on the directionality of the effect. Future research could elucidate the causality of unfavorable fatty acids profiles and the disruption of metabolic tissue circadian rhythms and, and the specific role of increased levels of saturated fatty acids for nightshift workers.

We observed no clear association for total cholesterol and nightshift work, which was in agreeance with one existing cross-sectional study including metabolic profiles (Huang *et al.*, 2021), but in contrast with two studies among male rotating shift workers: a cross-sectional study (Ghiasvand *et al.*, 2006) and a prospective study (34). We observed lower HDL levels in nightshift workers compared to day workers, which was in accordance with previous reports (Ghiasvand *et al.*, 2006; De Bacquer *et al.*, 2009; Alefishat and Abu Farha, 2015). In the NHSII population, a large study of female nurses, HDL cholesterol was associated with nightshift work, but these reduced HDL levels could not be confirmed in the group with the highest exposure; working five or more night shifts in the past two weeks (Johnson *et al.*, 2020). Although the effect of nightshift work on cholesterol and triglycerides has been studied more frequently, our approach with a broader metabolic profile is a new approach, allowing us to research subtypes. Our results were mainly driven by the large particle sizes of HDL, for which an inverse association with myocardial infarction has been reported (Holmes *et al.*, 2018). We observed consistently elevated VLDL triglycerides, especially amongst recently started nightshift workers. These results replicate findings from previous studies that showed an association between nightshift work and higher triglycerides levels (Ghiasvand *et al.*, 2006; De Bacquer *et al.*, 2009). However, some other studies do not confirm this association between nightshift work and triglyceride levels (Esquirol *et al.*, 2011; Guo *et al.*, 2015; Proper *et al.*, 2016; Huang *et al.*, 2021), which might be partly explained by the different age range of these study populations. We mainly find an effect in the (younger) recently started nightshift workers, while, for example, a large high quality cohort study in an older population of retired workers did not observe an effect in triglyceride levels (Guo *et al.*, 2015). Recently started nightshift workers had a mean age of 30 (SD 10), compared to a mean age 42 (SD 11) for the more experienced nightshift workers, which might have had an impact on differences in dietary intake and patterns between those groups. We made an effort to adjust for these differences by adding saturated fat intake and meal timing in the subset for which we had more elaborate covariates available (leading to similar results as the main analyses).

Several amino acids (histidine, glutamine, leucine, and isoleucine) were somewhat elevated in our study, especially among recently started nightshift workers. Leucine and isoleucine are branched-chain amino acids that are associated with insulin resistance: higher levels of these amino acids are observed in the blood of diabetic humans (Lynch and Adams, 2014). However, these effects of insulin resistance are not clear cut, in healthy humans, the combined amino acids leucine, isoleucine, and valine acutely elevate circulating insulin levels and enhance glucose clearance (Yang *et al.*, 2010).

### Strengths and weaknesses

The results of the current study apply to female nightshift workers employed in the hospital (nurses and paramedic staff). The rotating nightshift workers and control subject worked in similar professions and in a similar working environment, to limit confounding factors related to job exposure. Since the results apply to a specific population, the results cannot be directly extrapolated to other populations of nightshift workers, including males and other professions. The comparability between the groups was also increased by recruiting our control group of non-nightshift workers from the same hospitals as the rotating nightshift workers. Within our study, observed effects on lipoproteins, amino acids and fatty acids were similar or more pronounced among recently started (<2 years) nightshift workers as compared to the whole study population. This could indicate that the time span of previous nightshift work is relevant and that the effects manifest themselves more distinctly in recently started nightshift workers. However, our study, being cross-sectional in design, makes it not possible to assert causality. Furthermore, our study is prone to healthy-worker effect bias. Long-term exposure to nightshift work could lead to adaption to its disruptive effects or to self-selection of individuals that are better capable to withstand its disruptive effects. Adaptation and self-selection would manifest themselves in a similar way (e.g. fast adaptation of the circadian rhythm or fast re-synchronization). With our current study design, it is not possible to disentangle the effects of adaptation versus self-selection as a longitudinal study design would be needed. We selected a relatively healthy study population (non-smoking female nurses), since we excluded subjects that had high blood pressure (used beta-blockers); or diagnosed cardiovascular disease; or medication for type II diabetes. So, our results might be dampened by not including these pathologies.

Our study is observational in nature, assessing biomarkers during real working days of nurses and paramedic staff. As a consequence, the day workers may still have experienced circadian disorder, since working morning shifts and afternoon shifts can still be considered as irregular and could negatively impact blood lipids (Thomas and Power, 2010). Therefore, the power to find an effect in our study could be reduced. In our study, elevated levels of amino acids as a result from regularly working nightshifts were in contrast with the acute effects we have observed measured directly after a nightshift. A possible explanation is that amino acids have been identified as rhythmic metabolites in previous studies (Wurtman *et al.*, 1968; Feigin *et al.*, 1971; Skene *et al.*, 2018; Kervezee *et al.*, 2019). The same applies to lipoproteins, which also have an intrinsic circadian rhythmicity (Romon *et al.*, 1997). Although results were adjusted for the timing the blood sample was taken to take diurnal variation into account (after which the association for the amino acids becomes close to zero), residual confounding cannot be ruled out. The observational nature of our study also implies we used non-fasting blood samples, which might have had an influence on this difference in results, since fasting influences lipid levels (Umakanth and Ibrahim, 2018; Wefers *et al.*, 2018) and amino acids (Scheer *et al.*, 2009; Newman and Verdin, 2014; Morris *et al.*, 2015; Wefers *et al.*, 2018). Leucine levels and lactate levels were decreased directly after the nightshift, and levels of β-hydroxybutyrate were elevated, which is indicating a fastened state. We know from our previous study (van de Langenberg *et al.*, 2018) participants often eat less during a night shift. Therefore, we made an effort to adjust for timing of last meal before blood draw, and in our study, this turned out to be a confounding factor in the association between nightshift work and metabolic markers. This can be a consideration for future studies.

Our study provides new information on the associations between nightshift work and blood metabolites. Our study is one of the first studies to investigate a possible link between nightshift work and such a wide range of markers. Our study contributes to the existing evidence that the disruption of circadian rhythmicity, as induced by nightshift work, is a risk factor for obesity and metabolic disorders by demonstrating impaired lipid metabolism among night shift workers. The association between nightshift work and unfavorable patterns in fatty acid profiles was generally more pronounced in samples collected directly after the nightshift and among recently started compared to more experienced nightshift workers.

## Supplementary Material

wxad018_suppl_Supplementary_Material

## Data Availability

The data underlying this article will be shared on reasonable request to the corresponding author.
