# Supplementary material for: Effects of Nightshift Work on Blood Metabolites in Female Nurses and Paramedic Staff: A Cross-sectional Study
Source: Ann Work Expo Health. 2023 Apr 26;67(6):694–705. doi: 10.1093/annweh/wxad018 (PMC10394501; doi:10.1093/annweh/wxad018)
Supplement: wxad018_suppl_Supplementary_Material [file wxad018_suppl_supplementary_material.docx]

**Supplementary material**

Effects of nightshift work on blood metabolites in female nurses and paramedic staff: a cross-sectional study

*Daniella van de Langenberg^a/b^, Martijn E.T. Dollé ^b^, Linda W.M. van Kerkhof ^b^, and Roel C.H. Vermeulen^a^_,_ Jelle J. Vlaanderen^a*^*

**S1 Appendix. General characteristics of the study population when divided into the groups: day workers, recent nightshift workers, and experienced nightshift workers**

| *Table 1:* General characteristics of study population (n = 237, total number of observations = 361)^a^ | | | |
| --- | --- | --- | --- |
|  | Day workers (n=94) | Recent nightshift workers (n=36) | Experienced nightshift workers (n=107) |
| Number of samples/observations | 114 day-shift samples | 43 day-shift samples, 14 nightshift samples | 138 day-shift samples, 52 nightshift samples |
| Age in years (mean ± SD) | 44 ± 13 | 30 ± 10 | 42 ± 11 |
| Body mass index (mean ± SD)^b^ | 24.9 ± 4.2 | 23.2 ± 3.9 | 24.6 ± 4.1 |
| Years of nightshift work experience (mean ± SD) | - | 1.3 ± 0.8 | 18.0 ± 10.9 |
| *Chronotype* |  |  |  |
| Clearly morning person | 24.1% | 15.8% | 11.3% |
| More morning than evening person | 34.1% | 18.4% | 30.2% |
| No preference | 14.3% | 15.8% | 19.8% |
| More evening than morning person | 19.8% | 31.6% | 22.6% |
| Clearly evening person | 7.7% | 18.4% | 16.0% |
| *^a^ Missing data excluded from frequency table*  *^b^ Weight in kilograms divided by height in meters squared* | | | |

**S2 Appendix. Description of other than the first two PCA components and associations and scree plots of the eigenvalues. Nightshift workers compared to day workers**

Fatty acids

Component 3 was characterized by negative loadings for docosahexaenoic acid (DHA) and DHA/FA and ratio of omega-3 fatty acids to total fatty acids (FAw3/FA), and positive loadings for linoleic acid (LA) and FAw6. No associations were observed for component 3.

Amino acids

Component 3 was characterized by negative loadings for glucose and citrate, and, to a lesser degree lactate and alanine. Component 4 was characterized by positive loadings for lactate and negative loadings for glutamine and histidine. Component 5 was characterized by positive loadings for glucose and acetate, and negative loadings for lactate, alanine glutamine, and β-hydroxybutyrate. No statistically significant associations were observed for components 3, 4 and 5.

**Fig 1** Scree plots of the eigenvalues and variance explained (number of dimensions >70%)

**Fig 2** Principal component loading plots (blue squares indicate positive loading, whereas red (striped) squares indicate negative loadings for the complete set. Nightshift workers compared to day-workers

Abbreviations: TotCho = Total Cholesterol, C = Cholesterol, VLDL = very low density lipoprotein, LDL = low density lipoprotein, IDL = intermediate density lipoprotein, HDL = high-density lipoprotein (HDL2 particle density 1.063-1.125 g/mL, HDL3 1.125-1.210 g/mL), XL = extra-large, L= large, M = medium, S= small, TG = triglycerides, TotPG = Total phosphoglycerides, TotFA = Total fatty acids, FAw3 = Omega-3 fatty acids, FAw6 = Omega-6 fatty acids, PUFA = poly-unsaturated fatty acids, MUFA = mono-unsaturated fatty acids, SFA = saturated fatty acids, LA = linoleic acid, DHA = docosahexaenoic acid, LA.FA = Ratio of 18:2 linoleic acid to total fatty acids, DHA.FA = Ratio of 22:6 docosahexaenoic acid to total fatty acids, Ala = alanine, Gln = glutamine, His = histidine, Ile = isoleucine, Leu = leucine, Val = valine, Phe = phenylalanine, Tyr = tyrosine, Glc = glucose, Lac = Lactate, Cit = citrate, Ace = acetate, AcAce = acetoacetate, bOHBut = β-hydroxybutyrate, ApoA1 = apolipoprotein A-I, ApoB = apolipoprotein B, ApoB.ApoA1 = ratio of apolipoprotein B to apolipoprotein A-I

**S3a Appendix. Visual display of univariate analyses (n=237).** **Nightshift workers compared to day-workers on metabolic biomarkers**

**Fig 1** Univariate mixed-effect linear regression models (confidence intervals). Associations for long-term nightshift work and metabolites. Non-fasted blood samples nightshift workers (both recent and experienced, n=143) were compared to those of day workers (n=94). 1 SD increment in biomarker concentration. A = minimal confounder model, associations are adjusted for age. B = confounder model, associations are adjusted for age, and BMI. C = full-covariate model, associations are adjusted for age, BMI, chronotype, and blood sampling time. Abbreviations: TotCho = Total Cholesterol, C = Cholesterol, VLDL = very low density lipoprotein, LDL = low density lipoprotein, IDL = intermediate density lipoprotein, HDL = high-density lipoprotein (HDL2 particle density 1.063-1.125 g/mL, HDL3 1.125-1.210 g/mL), XL = extra-large, L= large, M = medium, S= small, TG = triglycerides, TotPG = Total phosphoglycerides, TotFA = Total fatty acids, FAw3 = Omega-3 fatty acids, FAw6 = Omega-6 fatty acids, PUFA = poly-unsaturated fatty acids, MUFA = mono-unsaturated fatty acids, SFA = saturated fatty acids, LA = linoleic acid, DHA = docosahexaenoic acid, LA.FA = Ratio of 18:2 linoleic acid to total fatty acids, DHA.FA = Ratio of 22:6 docosahexaenoic acid to total fatty acids, Ala = alanine, Gln = glutamine, His = histidine, Ile = isoleucine, Leu = leucine, Val = valine, Phe = phenylalanine, Tyr = tyrosine, Glc = glucose, Lac = Lactate, Cit = citrate, Ace = acetate, AcAce = acetoacetate, bOHBut = β-hydroxybutyrate, ApoA1 = apolipoprotein A-I, ApoB = apolipoprotein B, ApoB.ApoA1 = ratio of apolipoprotein B to apolipoprotein A-I

**S3b Appendix. Sensitivity analyses among the elaborate subset (n=90 individuals, 161 samples). Night-shift workers compared to day-workers**

**Fig 1** Associations for long-term night-shift work and metabolites. Non-fasted blood samples of night-shift workers (both recent and experienced, n=69, 121 samples measured during a day session) were compared to those of day workers (n=21, 40 samples). 1 SD increment in biomarker concentration (z-scores). A = minimal confounder model, associations are adjusted for age only, B = confounder model, associations are adjusted for age, BMI, and blood sampling time. C = full-covariate model, associations are adjusted for age, BMI, chronotype, timing of last meal before blood draw, saturated fat intake and blood sampling time. Abbreviations: TotCho = Total Cholesterol, C = Cholesterol, VLDL = very low density lipoprotein, LDL = low density lipoprotein, IDL = intermediate density lipoprotein, HDL = high-density lipoprotein (HDL2 particle density 1.063-1.125 g/mL, HDL3 1.125-1.210 g/mL), XL = extra-large, L= large, M = medium, S= small, TG = triglycerides, TotPG = Total phosphoglycerides, TotFA = Total fatty acids, FAw3 = Omega-3 fatty acids, FAw6 = Omega-6 fatty acids, PUFA = poly-unsaturated fatty acids, MUFA = mono-unsaturated fatty acids, SFA = saturated fatty acids, LA = linoleic acid, DHA = docosahexaenoic acid, LA.FA = Ratio of 18:2 linoleic acid to total fatty acids, DHA.FA = Ratio of 22:6 docosahexaenoic acid to total fatty acids, Ala = alanine, Gln = glutamine, His = histidine, Ile = isoleucine, Leu = leucine, Val = valine, Phe = phenylalanine, Tyr = tyrosine, Glc = glucose, Lac = Lactate, Cit = citrate, Ace = acetate, AcAce = acetoacetate, bOHBut = β-hydroxybutyrate, ApoA1 = apolipoprotein A-I, ApoB = apolipoprotein B, ApoB.ApoA1 = ratio of apolipoprotein B to apolipoprotein A-I

**S4 Appendix. Experienced nightshift workers (>5 years) and recently started nightshift workers (<2 years) compared to day workers (n=237 individuals). PCA and univariate analyses**

**Fig 1** PCA loading plots and associations of linear regression analyses (confidence intervals) between nightshift work and components. Left part: Principal component loading plots (blue squares indicate positive loading, whereas red (striped) squares indicate negative loadings. Right part: associations between status of nightshift work and metabolites. Blood samples drawn during a non-night shift of recently started nightshift workers (<2 years, n = 36) and experienced nightshift workers (>5 years, n = 107) were compared to those of day workers (n = 94). Full-covariate model, associations are adjusted for age, BMI, chronotype, and blood sampling time. Abbreviations: TotCho = Total Cholesterol, C = Cholesterol, VLDL = very low density lipoprotein, LDL = low density lipoprotein, IDL = intermediate density lipoprotein, HDL = high-density lipoprotein (HDL2 particle density 1.063-1.125 g/mL, HDL3 1.125-1.210 g/mL), XL = extra-large, L= large, M = medium, S= small, TG = triglycerides, TotPG = Total phosphoglycerides, TotFA = Total fatty acids, FAw3 = Omega-3 fatty acids, FAw6 = Omega-6 fatty acids, PUFA = poly-unsaturated fatty acids, MUFA = mono-unsaturated fatty acids, SFA = saturated fatty acids, LA = linoleic acid, DHA = docosahexaenoic acid, LA.FA = Ratio of 18:2 linoleic acid to total fatty acids, DHA.FA = Ratio of 22:6 docosahexaenoic acid to total fatty acids, Ala = alanine, Gln = glutamine, His = histidine, Ile = isoleucine, Leu = leucine, Val = valine, Phe = phenylalanine, Tyr = tyrosine, Glc = glucose, Lac = Lactate, Cit = citrate, Ace = acetate, AcAce = acetoacetate, bOHBut = β-hydroxybutyrate, ApoA1 = apolipoprotein A-I, ApoB = apolipoprotein B, ApoB.ApoA1 = ratio of apolipoprotein B to apolipoprotein A-I

**Fig 2** Mixed-effect linear regression models. Associations between status of nightshift work and metabolites. Non-fasted blood samples drawn during a non-night shift of recently started nightshift workers (<2 years, n = 36) and experienced nightshift workers (>5 years, n = 107) were compared to those of day workers (n = 94). 1 SD increment in biomarker concentration (z-scores). Full-covariate model, associations are adjusted for age, BMI, chronotype, and blood sampling time. Abbreviations: TotCho = Total Cholesterol, C = Cholesterol, VLDL = very low density lipoprotein, LDL = low density lipoprotein, IDL = intermediate density lipoprotein, HDL = high-density lipoprotein (HDL2 particle density 1.063-1.125 g/mL, HDL3 1.125-1.210 g/mL), XL = extra-large, L= large, M = medium, S= small, TG = triglycerides, TotPG = Total phosphoglycerides, TotFA = Total fatty acids, FAw3 = Omega-3 fatty acids, FAw6 = Omega-6 fatty acids, PUFA = poly-unsaturated fatty acids, MUFA = mono-unsaturated fatty acids, SFA = saturated fatty acids, LA = linoleic acid, DHA = docosahexaenoic acid, LA.FA = Ratio of 18:2 linoleic acid to total fatty acids, DHA.FA = Ratio of 22:6 docosahexaenoic acid to total fatty acids, Ala = alanine, Gln = glutamine, His = histidine, Ile = isoleucine, Leu = leucine, Val = valine, Phe = phenylalanine, Tyr = tyrosine, Glc = glucose, Lac = Lactate, Cit = citrate, Ace = acetate, AcAce = acetoacetate, bOHBut = β-hydroxybutyrate, ApoA1 = apolipoprotein A-I, ApoB = apolipoprotein B, ApoB.ApoA1 = ratio of apolipoprotein B to apolipoprotein A-I

**S5 Appendix. Description of other than the first two PCA components and associations and scree plots of the eigenvalues. Acute effects measured directly after a nightshift**

Amino acids

Component 1 to 4 of the amino acids had a similar loading pattern as described previously, within the nightshift workers compared to day workers. Component 5 was characterized by negative loadings for glucose and acetate, and positive loadings for lactate, citrate and alanine, which was in contrast with the loadings described within the nightshift workers compared to day workers analyses. Component 3, primarily negatively loaded by citrate, alanine, glutamine and acetate, was significantly elevated after working a night shift in model A and B. This association resulted in a null-effect in the full-covariate model. We observed no association for component 4. Component 5 (characterized by negative loadings for glucose and acetate, and positive loadings for lactate, citrate and alanine) was consistently decreased (β -0.58, BI -1.26, 0.10, Fig 2).

**Fig 1** Scree plots of the eigenvalues and variance explained (number of dimensions >70%)

**Fig 2** Principal component loading plots for the complete set (blue squares indicate positive loading, whereas red (striped) squares indicate negative loadings. Acute effects measured directly after a nightshift

Abbreviations: TotCho = Total Cholesterol, C = Cholesterol, VLDL = very low density lipoprotein, LDL = low density lipoprotein, IDL = intermediate density lipoprotein, HDL = high-density lipoprotein (HDL2 particle density 1.063-1.125 g/mL, HDL3 1.125-1.210 g/mL), XL = extra-large, L= large, M = medium, S= small, TG = triglycerides, TotPG = Total phosphoglycerides, TotFA = Total fatty acids, FAw3 = Omega-3 fatty acids, FAw6 = Omega-6 fatty acids, PUFA = poly-unsaturated fatty acids, MUFA = mono-unsaturated fatty acids, SFA = saturated fatty acids, LA = linoleic acid, DHA = docosahexaenoic acid, LA.FA = Ratio of 18:2 linoleic acid to total fatty acids, DHA.FA = Ratio of 22:6 docosahexaenoic acid to total fatty acids, Ala = alanine, Gln = glutamine, His = histidine, Ile = isoleucine, Leu = leucine, Val = valine, Phe = phenylalanine, Tyr = tyrosine, Glc = glucose, Lac = Lactate, Cit = citrate, Ace = acetate, AcAce = acetoacetate, bOHBut = β-hydroxybutyrate, ApoA1 = apolipoprotein A-I, ApoB = apolipoprotein B, ApoB.ApoA1 = ratio of apolipoprotein B to apolipoprotein A-I

Component 1 of the complete set of biomarkers was characterized by negative loadings for all lipoproteins except for the large particle sizes of HDL. Component 2 is characterized by negative VLDL loadings (all but the XS particle size) and positive LDL and HDL lipoproteins.

**S6 Appendix. Univariate analyses (n=237).** **Linear regression models for chronic and acute effects of shift work and descriptive statistics of all 225 metabolite measures.**

| **Supplementary table I. Descriptive statistics of metabolite measures of 237 female nurses and paramedic staff blood samples.** | | | | |
| --- | --- | --- | --- | --- |
| Metabolite (units are in mmol/l unless stated otherwise) | Mean | Median | Standard deviation | Standard error |
| XXL.VLDL.P (mol/l) | 1.25E-10 | 9.76E-11 | 1.13E-10 | 5.87E-12 |
| XXL.VLDL.L | 0.026269 | 0.0204 | 0.024331 | 0.001253 |
| XXL.VLDL.PL | 0.002929 | 0.00214 | 0.003028 | 0.000156 |
| XXL.VLDL.C | 0.00396 | 0.00288 | 0.003963 | 0.000204 |
| XXL.VLDL.CE | 0.002358 | 0.00184 | 0.002179 | 0.000112 |
| XXL.VLDL.FC | 0.001602 | 0.00103 | 0.001845 | 9.50E-05 |
| XXL.VLDL.TG | 0.019345 | 0.0154 | 0.017894 | 0.000922 |
| XL.VLDL.P (mol/l) | 5.29E-10 | 3.69E-10 | 5.90E-10 | 3.07E-11 |
| XL.VLDL.L | 0.052005 | 0.0355 | 0.059019 | 0.00304 |
| XL.VLDL.PL | 0.00833 | 0.00521 | 0.009758 | 0.000503 |
| XL.VLDL.C | 0.010546 | 0.00703 | 0.011783 | 0.000607 |
| XL.VLDL.CE | 0.005647 | 0.00397 | 0.005941 | 0.000306 |
| XL.VLDL.FC | 0.004526 | 0.00282 | 0.005411 | 0.000279 |
| XL.VLDL.TG | 0.033244 | 0.0231 | 0.038012 | 0.001958 |
| L.VLDL.P (mol/l) | 3.33E-09 | 2.49E-09 | 3.08E-09 | 1.60E-10 |
| L.VLDL.L | 0.197566 | 0.145 | 0.192414 | 0.00991 |
| L.VLDL.PL | 0.035084 | 0.0251 | 0.033996 | 0.001751 |
| L.VLDL.C | 0.043098 | 0.0327 | 0.041481 | 0.002136 |
| L.VLDL.CE | 0.024739 | 0.0196 | 0.02165 | 0.001115 |
| L.VLDL.FC | 0.018458 | 0.0123 | 0.020788 | 0.001071 |
| L.VLDL.TG | 0.116153 | 0.0876 | 0.108256 | 0.005575 |
| M.VLDL.P (mol/l) | 1.29E-08 | 1.10E-08 | 7.65E-09 | 3.98E-10 |
| M.VLDL.L | 0.434468 | 0.369 | 0.26644 | 0.013722 |
| M.VLDL.PL | 0.087663 | 0.076 | 0.049726 | 0.002561 |
| M.VLDL.C | 0.117262 | 0.102 | 0.067408 | 0.003472 |
| M.VLDL.CE | 0.071974 | 0.0646 | 0.037792 | 0.001946 |
| M.VLDL.FC | 0.046021 | 0.0385 | 0.031979 | 0.001647 |
| M.VLDL.TG | 0.22486 | 0.192 | 0.141045 | 0.007264 |
| S.VLDL.P (mol/l) | 2.25E-08 | 2.12E-08 | 9.01E-09 | 4.69E-10 |
| S.VLDL.L | 0.437496 | 0.414 | 0.175796 | 0.009054 |
| S.VLDL.PL | 0.106077 | 0.0997 | 0.03859 | 0.001988 |
| S.VLDL.C | 0.155625 | 0.15 | 0.065516 | 0.003374 |
| S.VLDL.CE | 0.09716 | 0.0927 | 0.042853 | 0.002207 |
| S.VLDL.FC | 0.059252 | 0.0547 | 0.024432 | 0.001258 |
| S.VLDL.TG | 0.179434 | 0.162 | 0.07917 | 0.004077 |
| XS.VLDL.P (mol/l) | 3.40E-08 | 3.30E-08 | 8.90E-09 | 4.63E-10 |
| XS.VLDL.L | 0.433576 | 0.42 | 0.116394 | 0.005995 |
| XS.VLDL.PL | 0.131859 | 0.127 | 0.036448 | 0.001877 |
| XS.VLDL.C | 0.214805 | 0.21 | 0.05945 | 0.003062 |
| XS.VLDL.CE | 0.150338 | 0.147 | 0.042687 | 0.002199 |
| XS.VLDL.FC | 0.066068 | 0.0646 | 0.018968 | 0.000977 |
| XS.VLDL.TG | 0.08536 | 0.0796 | 0.027925 | 0.001438 |
| IDL.P (mol/l) | 1.01E-07 | 9.98E-08 | 2.43E-08 | 1.27E-09 |
| IDL.L | 1.035358 | 1.02 | 0.251966 | 0.012977 |
| IDL.PL | 0.283984 | 0.281 | 0.063333 | 0.003262 |
| IDL.C | 0.653074 | 0.642 | 0.172446 | 0.008881 |
| IDL.CE | 0.462233 | 0.455 | 0.124046 | 0.006389 |
| IDL.FC | 0.189047 | 0.187 | 0.048151 | 0.00248 |
| IDL.TG | 0.096273 | 0.0922 | 0.025424 | 0.001309 |
| L.LDL.P (mol/l) | 1.68E-07 | 1.65E-07 | 4.25E-08 | 2.21E-09 |
| L.LDL.L | 1.197024 | 1.18 | 0.306226 | 0.015771 |
| L.LDL.PL | 0.305618 | 0.303 | 0.064605 | 0.003327 |
| L.LDL.C | 0.80809 | 0.795 | 0.225895 | 0.011634 |
| L.LDL.CE | 0.575043 | 0.56 | 0.174078 | 0.008965 |
| L.LDL.FC | 0.235379 | 0.234 | 0.054533 | 0.002809 |
| L.LDL.TG | 0.086995 | 0.0831 | 0.023924 | 0.001232 |
| M.LDL.P (mol/l) | 1.36E-07 | 1.33E-07 | 3.64E-08 | 1.89E-09 |
| M.LDL.L | 0.693735 | 0.679 | 0.184276 | 0.009491 |
| M.LDL.PL | 0.185941 | 0.181 | 0.038297 | 0.001972 |
| M.LDL.C | 0.463523 | 0.454 | 0.140214 | 0.007221 |
| M.LDL.CE | 0.329791 | 0.321 | 0.114375 | 0.005954 |
| M.LDL.FC | 0.133376 | 0.131 | 0.026789 | 0.00138 |
| M.LDL.TG | 0.044718 | 0.0433 | 0.011985 | 0.000617 |
| S.LDL.P (mol/l) | 1.60E-07 | 1.56E-07 | 4.07E-08 | 2.12E-09 |
| S.LDL.L | 0.450647 | 0.44 | 0.114151 | 0.005879 |
| S.LDL.PL | 0.13856 | 0.138 | 0.02621 | 0.00135 |
| S.LDL.C | 0.282729 | 0.278 | 0.087329 | 0.004498 |
| S.LDL.CE | 0.202759 | 0.196 | 0.070469 | 0.003629 |
| S.LDL.FC | 0.082006 | 0.0808 | 0.016806 | 0.000866 |
| S.LDL.TG | 0.027339 | 0.0262 | 0.007883 | 0.000406 |
| XL.HDL.P (mol/l) | 5.73E-07 | 5.37E-07 | 2.10E-07 | 1.09E-08 |
| XL.HDL.L | 0.576793 | 0.541 | 0.216434 | 0.011147 |
| XL.HDL.PL | 0.281982 | 0.272 | 0.114793 | 0.005912 |
| XL.HDL.C | 0.280973 | 0.265 | 0.101074 | 0.005206 |
| XL.HDL.CE | 0.207218 | 0.197 | 0.07287 | 0.003753 |
| XL.HDL.FC | 0.074902 | 0.0701 | 0.029395 | 0.001514 |
| XL.HDL.TG | 0.017529 | 0.0164 | 0.00731 | 0.000376 |
| L.HDL.P (mol/l) | 1.37E-06 | 1.33E-06 | 4.78E-07 | 2.46E-08 |
| L.HDL.L | 0.866446 | 0.834 | 0.309961 | 0.016136 |
| L.HDL.PL | 0.411152 | 0.402 | 0.13403 | 0.006903 |
| L.HDL.C | 0.419897 | 0.405 | 0.169475 | 0.008728 |
| L.HDL.CE | 0.327884 | 0.316 | 0.1288 | 0.006705 |
| L.HDL.FC | 0.091767 | 0.0881 | 0.041336 | 0.002129 |
| L.HDL.TG | 0.032073 | 0.0299 | 0.014933 | 0.000769 |
| M.HDL.P (mol/l) | 2.01E-06 | 1.97E-06 | 3.41E-07 | 1.77E-08 |
| M.HDL.L | 0.850156 | 0.836 | 0.146699 | 0.007555 |
| M.HDL.PL | 0.401027 | 0.395 | 0.067835 | 0.003494 |
| M.HDL.C | 0.410788 | 0.404 | 0.080236 | 0.004132 |
| M.HDL.CE | 0.337013 | 0.331 | 0.062851 | 0.003237 |
| M.HDL.FC | 0.076187 | 0.0751 | 0.017667 | 0.00091 |
| M.HDL.TG | 0.039142 | 0.0368 | 0.013393 | 0.00069 |
| S.HDL.P (mol/l) | 4.64E-06 | 4.65E-06 | 5.27E-07 | 2.72E-08 |
| S.HDL.L | 1.028228 | 1.03 | 0.119371 | 0.006148 |
| S.HDL.PL | 0.568394 | 0.563 | 0.076795 | 0.003955 |
| S.HDL.C | 0.416577 | 0.416 | 0.063002 | 0.003245 |
| S.HDL.CE | 0.314227 | 0.313 | 0.057958 | 0.002985 |
| S.HDL.FC | 0.102652 | 0.102 | 0.013511 | 0.000696 |
| S.HDL.TG | 0.043207 | 0.0414 | 0.012059 | 0.000621 |
| XXL.VLDL.PL_. (ratio of total) | 9.59301 | 10.6 | 2.987972 | 0.153888 |
| XXL.VLDL.C_. (ratio of total) | 12.87549 | 14.3 | 6.638452 | 0.341898 |
| XXL.VLDL.CE_. (ratio of total) | 7.968199 | 8.87 | 4.526434 | 0.233123 |
| XXL.VLDL.FC_. (ratio of total) | 4.755126 | 5.39 | 2.399494 | 0.12358 |
| XXL.VLDL.TG_. (ratio of total) | 69.22405 | 73.5 | 15.89839 | 0.818809 |
| XL.VLDL.PL_. (ratio of total) | 12.9555 | 15.8 | 6.853423 | 0.352969 |
| XL.VLDL.C_. (ratio of total) | 18.09871 | 19.6 | 7.8673 | 0.405187 |
| XL.VLDL.CE_. (ratio of total) | 10.32909 | 10.8 | 6.409577 | 0.33011 |
| XL.VLDL.FC_. (ratio of total) | 7.019737 | 8.22 | 4.222719 | 0.217481 |
| XL.VLDL.TG_. (ratio of total) | 54.69248 | 62.8 | 20.91907 | 1.077387 |
| L.VLDL.PL_. (ratio of total) | 16.76935 | 17.7 | 2.647768 | 0.136367 |
| L.VLDL.C_. (ratio of total) | 20.2134 | 21.8 | 5.065135 | 0.260868 |
| L.VLDL.CE_. (ratio of total) | 12.30189 | 12.9 | 4.159829 | 0.214242 |
| L.VLDL.FC_. (ratio of total) | 7.105225 | 8.37 | 3.737181 | 0.192475 |
| L.VLDL.TG_. (ratio of total) | 57.60035 | 59.5 | 6.937166 | 0.357282 |
| M.VLDL.PL_. (ratio of total) | 20.77557 | 20.6 | 0.989906 | 0.050983 |
| M.VLDL.C_. (ratio of total) | 27.56954 | 27.3 | 4.648274 | 0.239398 |
| M.VLDL.CE_. (ratio of total) | 17.58234 | 16.9 | 4.678482 | 0.240954 |
| M.VLDL.FC_. (ratio of total) | 10.14116 | 10.5 | 1.742662 | 0.089752 |
| M.VLDL.TG_. (ratio of total) | 51.48033 | 52.1 | 4.92947 | 0.253881 |
| S.VLDL.PL_. (ratio of total) | 24.54124 | 24.2 | 1.877534 | 0.096698 |
| S.VLDL.C_. (ratio of total) | 35.26946 | 35.3 | 5.266444 | 0.271236 |
| S.VLDL.CE_. (ratio of total) | 21.9046 | 21.8 | 5.111402 | 0.263251 |
| S.VLDL.FC_. (ratio of total) | 13.35345 | 13.4 | 0.865293 | 0.044565 |
| S.VLDL.TG_. (ratio of total) | 40.26518 | 40.4 | 4.974524 | 0.256201 |
| XS.VLDL.PL_. (ratio of total) | 30.47772 | 30.6 | 2.346156 | 0.120833 |
| XS.VLDL.C_. (ratio of total) | 49.79867 | 50 | 3.007336 | 0.154886 |
| XS.VLDL.CE_. (ratio of total) | 34.57427 | 34.8 | 3.169444 | 0.163235 |
| XS.VLDL.FC_. (ratio of total) | 15.16801 | 15.4 | 1.325292 | 0.068256 |
| XS.VLDL.TG_. (ratio of total) | 19.86101 | 19.5 | 3.908909 | 0.201319 |
| IDL.PL_. (ratio of total) | 27.63952 | 27.6 | 0.862612 | 0.044427 |
| IDL.C_. (ratio of total) | 62.83156 | 63 | 2.234851 | 0.115101 |
| IDL.CE_. (ratio of total) | 44.54828 | 44.8 | 2.438718 | 0.1256 |
| IDL.FC_. (ratio of total) | 18.25178 | 18.5 | 1.351717 | 0.069617 |
| IDL.TG_. (ratio of total) | 9.54122 | 9.26 | 1.998046 | 0.102905 |
| L.LDL.PL_. (ratio of total) | 25.76499 | 25.5 | 1.441352 | 0.074233 |
| L.LDL.C_. (ratio of total) | 66.83342 | 67.2 | 2.682587 | 0.13816 |
| L.LDL.CE_. (ratio of total) | 47.10318 | 47.7 | 3.2769 | 0.168769 |
| L.LDL.FC_. (ratio of total) | 19.74403 | 19.7 | 1.088906 | 0.056081 |
| L.LDL.TG_. (ratio of total) | 7.408462 | 7.12 | 1.750566 | 0.090159 |
| M.LDL.PL_. (ratio of total) | 27.36578 | 26.8 | 2.965111 | 0.152711 |
| M.LDL.C_. (ratio of total) | 65.96366 | 66.8 | 4.164507 | 0.214483 |
| M.LDL.CE_. (ratio of total) | 46.27294 | 47.5 | 5.799414 | 0.298685 |
| M.LDL.FC_. (ratio of total) | 19.70716 | 19.3 | 1.963626 | 0.101132 |
| M.LDL.TG_. (ratio of total) | 6.662334 | 6.38 | 1.714024 | 0.088277 |
| S.LDL.PL_. (ratio of total) | 31.53714 | 30.8 | 3.514015 | 0.180981 |
| S.LDL.C_. (ratio of total) | 62.25809 | 63.2 | 4.540872 | 0.233867 |
| S.LDL.CE_. (ratio of total) | 43.81942 | 45 | 5.78133 | 0.297754 |
| S.LDL.FC_. (ratio of total) | 18.47241 | 18.2 | 1.591461 | 0.081964 |
| S.LDL.TG_. (ratio of total) | 6.219151 | 5.94 | 1.552174 | 0.079941 |
| XL.HDL.PL_. (ratio of total) | 47.97666 | 48.7 | 5.750059 | 0.296143 |
| XL.HDL.C_. (ratio of total) | 48.80796 | 48.3 | 4.945107 | 0.254686 |
| XL.HDL.CE_. (ratio of total) | 35.95862 | 35.5 | 4.664948 | 0.240257 |
| XL.HDL.FC_. (ratio of total) | 12.8765 | 13 | 0.842323 | 0.043382 |
| XL.HDL.TG_. (ratio of total) | 3.205483 | 2.91 | 1.411623 | 0.072702 |
| L.HDL.PL_. (ratio of total) | 48.68708 | 48.2 | 3.241593 | 0.166951 |
| L.HDL.C_. (ratio of total) | 47.37248 | 48.2 | 3.977173 | 0.204835 |
| L.HDL.CE_. (ratio of total) | 37.23751 | 37.8 | 2.522399 | 0.12991 |
| L.HDL.FC_. (ratio of total) | 10.10585 | 10.5 | 1.685301 | 0.086797 |
| L.HDL.TG_. (ratio of total) | 3.795106 | 3.55 | 1.436734 | 0.073996 |
| M.HDL.PL_. (ratio of total)v | 47.13554 | 47 | 1.217737 | 0.062717 |
| M.HDL.C_. (ratio of total) | 48.24642 | 48.5 | 2.076847 | 0.106963 |
| M.HDL.CE_. (ratio of total) | 39.38541 | 39.6 | 1.906756 | 0.098203 |
| M.HDL.FC_. (ratio of total) | 8.847905 | 8.97 | 0.756456 | 0.038959 |
| M.HDL.TG_. (ratio of total) | 4.636976 | 4.34 | 1.537572 | 0.079189 |
| S.HDL.PL_. (ratio of total) | 55.19274 | 55.2 | 3.507713 | 0.180656 |
| S.HDL.C_. (ratio of total) | 40.49654 | 40.7 | 4.131974 | 0.212807 |
| S.HDL.CE_. (ratio of total) | 30.55809 | 30.7 | 4.429183 | 0.228115 |
| S.HDL.FC_. (ratio of total) | 9.971233 | 9.9 | 0.612271 | 0.031534 |
| S.HDL.TG_. (ratio of total) | 4.187276 | 4.02 | 1.075031 | 0.055367 |
| VLDL.D (nm) | 36.31592 | 36.2 | 1.412392 | 0.072742 |
| LDL.D (nm) | 23.53581 | 23.5 | 0.103997 | 0.005356 |
| HDL.D (nm) | 10.13143 | 10.1 | 0.258548 | 0.013316 |
| Serum.C | 4.27748 | 4.21 | 0.845845 | 0.043563 |
| VLDL.C | 0.549271 | 0.508 | 0.225895 | 0.011634 |
| Remnant.C | 1.197244 | 1.16 | 0.360386 | 0.018561 |
| LDL.C | 1.551149 | 1.52 | 0.448821 | 0.023115 |
| HDL.C | 1.532167 | 1.51 | 0.29925 | 0.015412 |
| HDL2.C | 1.039992 | 1.02 | 0.276385 | 0.014235 |
| HDL3.C | 0.490061 | 0.489 | 0.029518 | 0.00152 |
| EstC | 3.008859 | 2.97 | 0.611905 | 0.031515 |
| FreeC | 1.277828 | 1.27 | 0.249854 | 0.012868 |
| Serum.TG | 1.040504 | 0.927 | 0.452203 | 0.02329 |
| VLDL.TG | 0.654523 | 0.55 | 0.403129 | 0.020762 |
| LDL.TG | 0.16029 | 0.154 | 0.044645 | 0.002299 |
| HDL.TG | 0.131889 | 0.126 | 0.034369 | 0.00177 |
| TotPG | 1.992653 | 1.96 | 0.346445 | 0.017843 |
| TG.PG | 0.506523 | 0.472 | 0.2207 | 0.011367 |
| PC | 2.017878 | 1.99 | 0.340582 | 0.017541 |
| SM | 0.442615 | 0.437 | 0.071493 | 0.003682 |
| TotCho | 2.451167 | 2.43 | 0.36291 | 0.018691 |
| ApoA1 (g/l) | 1.544509 | 1.53 | 0.180684 | 0.009306 |
| ApoB (g/l) | 0.802939 | 0.787 | 0.181615 | 0.009354 |
| ApoB.ApoA1 (ratio) | 0.526838 | 0.506 | 0.119559 | 0.006158 |
| TotFA | 10.77308 | 10.5 | 2.326104 | 0.1198 |
| UnSat | 1.17644 | 1.18 | 0.056727 | 0.002922 |
| DHA | 0.099059 | 0.0952 | 0.037162 | 0.001914 |
| LA | 2.792308 | 2.74 | 0.559896 | 0.028836 |
| FAw3 | 0.37126 | 0.358 | 0.115672 | 0.005957 |
| FAw6 | 3.495729 | 3.45 | 0.646065 | 0.033274 |
| PUFA | 3.852387 | 3.8 | 0.72005 | 0.037084 |
| MUFA | 2.944058 | 2.78 | 0.804714 | 0.041445 |
| SFA | 3.961645 | 3.83 | 0.893384 | 0.046012 |
| DHA.FA (ratio of total) | 0.921093 | 0.912 | 0.282333 | 0.014541 |
| LA.FA (ratio of total) | 26.1756 | 26.4 | 2.651673 | 0.136568 |
| FAw3.FA (ratio of total) | 3.455862 | 3.4 | 0.716248 | 0.036889 |
| FAw6.FA (ratio of total) | 32.5992 | 33 | 2.685149 | 0.138292 |
| PUFA.FA (ratio of total) | 36.08011 | 36.5 | 2.764459 | 0.142377 |
| MUFA.FA (ratio of total) | 27.22679 | 27 | 2.454793 | 0.126428 |
| SFA.FA (ratio of total) | 36.69204 | 36.7 | 1.483398 | 0.076399 |
| Glc | 4.08008 | 3.98 | 0.713212 | 0.036732 |
| Lac | 1.122082 | 1.07 | 0.306035 | 0.015762 |
| Cit | 0.135679 | 0.134 | 0.022918 | 0.00118 |
| Ala | 0.383024 | 0.376 | 0.061537 | 0.003169 |
| Gln | 0.456629 | 0.459 | 0.073897 | 0.003806 |
| His | 0.060416 | 0.0599 | 0.009401 | 0.000484 |
| Ile | 0.056911 | 0.054 | 0.020931 | 0.001078 |
| Leu | 0.072641 | 0.0691 | 0.024866 | 0.001281 |
| Val | 0.159496 | 0.155 | 0.042506 | 0.002189 |
| Phe | 0.066211 | 0.0643 | 0.012399 | 0.000639 |
| Tyr | 0.059114 | 0.0562 | 0.019482 | 0.001003 |
| Ace | 0.049623 | 0.0464 | 0.015115 | 0.000778 |
| AcAce | 0.047538 | 0.0402 | 0.028795 | 0.001483 |
| bOHBut | 0.123916 | 0.107 | 0.063197 | 0.003255 |
| Crea | 0.05318 | 0.0529 | 0.007117 | 0.000367 |
| Alb | 0.08688 | 0.0868 | 0.004754 | 0.000245 |
| Gp | 1.197318 | 1.16 | 0.215531 | 0.0111 |

| **Supplementary table II. Estimates and standard errors for metabolite associations of short term (recent, <2 years) and long-term (>5 years) shift workers vs controls (workers that did not work night shifts). univariate linear regression models.** | | | | |
| --- | --- | --- | --- | --- |
| Metabolite | Trait | Estimates | Standard deviations | p-values |
| XXL.VLDL.P | shift work: < 2 years | 0.105661 | 0.224461 | 0.638339 |
| XXL.VLDL.L | shift work: < 2 years | 0.110967 | 0.228661 | 0.62799 |
| XXL.VLDL.PL | shift work: < 2 years | 0.159685 | 0.272813 | 0.559004 |
| XXL.VLDL.C | shift work: < 2 years | 0.001282 | 0.357659 | 0.997145 |
| XXL.VLDL.CE | shift work: < 2 years | 0.00966 | 0.460551 | 0.983287 |
| XXL.VLDL.FC | shift work: < 2 years | 0.145962 | 0.344967 | 0.672679 |
| XXL.VLDL.TG | shift work: < 2 years | 0.182125 | 0.215594 | 0.39928 |
| XL.VLDL.P | shift work: < 2 years | 0.085924 | 0.533553 | 0.872226 |
| XL.VLDL.L | shift work: < 2 years | 0.092675 | 0.524993 | 0.86007 |
| XL.VLDL.PL | shift work: < 2 years | -0.12319 | 0.845101 | 0.884251 |
| XL.VLDL.C | shift work: < 2 years | -0.00456 | 0.624564 | 0.994179 |
| XL.VLDL.CE | shift work: < 2 years | -0.0851 | 0.728385 | 0.907105 |
| XL.VLDL.FC | shift work: < 2 years | -0.0357 | 0.62837 | 0.954752 |
| XL.VLDL.TG | shift work: < 2 years | 0.033289 | 0.516177 | 0.948642 |
| L.VLDL.P | shift work: < 2 years | 0.033118 | 0.225817 | 0.883555 |
| L.VLDL.L | shift work: < 2 years | 0.126086 | 0.231181 | 0.586082 |
| L.VLDL.PL | shift work: < 2 years | 0.040858 | 0.21955 | 0.852562 |
| L.VLDL.C | shift work: < 2 years | 0.035568 | 0.232408 | 0.878524 |
| L.VLDL.CE | shift work: < 2 years | -0.20784 | 0.228335 | 0.363832 |
| L.VLDL.FC | shift work: < 2 years | -0.03062 | 0.421287 | 0.942135 |
| L.VLDL.TG | shift work: < 2 years | 0.153585 | 0.225059 | 0.495786 |
| M.VLDL.P | shift work: < 2 years | 0.017331 | 0.123709 | 0.888729 |
| M.VLDL.L | shift work: < 2 years | 0.095152 | 0.127374 | 0.455938 |
| M.VLDL.PL | shift work: < 2 years | 0.02813 | 0.118941 | 0.813284 |
| M.VLDL.C | shift work: < 2 years | -0.05824 | 0.14544 | 0.689256 |
| M.VLDL.CE | shift work: < 2 years | 0.033258 | 0.135819 | 0.806812 |
| M.VLDL.FC | shift work: < 2 years | 0.047413 | 0.1753 | 0.787071 |
| M.VLDL.TG | shift work: < 2 years | 0.059648 | 0.128972 | 0.644236 |
| S.VLDL.P | shift work: < 2 years | -0.03471 | 0.085112 | 0.683874 |
| S.VLDL.L | shift work: < 2 years | -0.05896 | 0.084582 | 0.486592 |
| S.VLDL.PL | shift work: < 2 years | -0.01802 | 0.075151 | 0.810788 |
| S.VLDL.C | shift work: < 2 years | -0.13276 | 0.09965 | 0.184321 |
| S.VLDL.CE | shift work: < 2 years | -0.08929 | 0.131672 | 0.498452 |
| S.VLDL.FC | shift work: < 2 years | -0.07587 | 0.089989 | 0.400213 |
| S.VLDL.TG | shift work: < 2 years | 0.013132 | 0.092294 | 0.886999 |
| XS.VLDL.P | shift work: < 2 years | -0.03211 | 0.055801 | 0.565669 |
| XS.VLDL.L | shift work: < 2 years | -0.02971 | 0.055538 | 0.593337 |
| XS.VLDL.PL | shift work: < 2 years | -0.05391 | 0.059659 | 0.367288 |
| XS.VLDL.C | shift work: < 2 years | -0.00442 | 0.059619 | 0.940997 |
| XS.VLDL.CE | shift work: < 2 years | 0.003412 | 0.060759 | 0.955277 |
| XS.VLDL.FC | shift work: < 2 years | -0.06084 | 0.063772 | 0.341222 |
| XS.VLDL.TG | shift work: < 2 years | 0.013046 | 0.066915 | 0.845628 |
| IDL.P | shift work: < 2 years | -0.03845 | 0.053707 | 0.474955 |
| IDL.L | shift work: < 2 years | -0.03446 | 0.053983 | 0.524026 |
| IDL.PL | shift work: < 2 years | -0.03573 | 0.050198 | 0.477473 |
| IDL.C | shift work: < 2 years | -0.05113 | 0.058799 | 0.385551 |
| IDL.CE | shift work: < 2 years | -0.03198 | 0.060053 | 0.594934 |
| IDL.FC | shift work: < 2 years | -0.06858 | 0.060114 | 0.255309 |
| IDL.TG | shift work: < 2 years | 0.001846 | 0.057117 | 0.974257 |
| L.LDL.P | shift work: < 2 years | -0.05309 | 0.057382 | 0.355996 |
| L.LDL.L | shift work: < 2 years | -0.0649 | 0.057392 | 0.259512 |
| L.LDL.PL | shift work: < 2 years | -0.04205 | 0.046096 | 0.362822 |
| L.LDL.C | shift work: < 2 years | -0.06831 | 0.064574 | 0.291419 |
| L.LDL.CE | shift work: < 2 years | -0.07415 | 0.070877 | 0.29674 |
| L.LDL.FC | shift work: < 2 years | -0.06808 | 0.053229 | 0.202431 |
| L.LDL.TG | shift work: < 2 years | -0.03402 | 0.059881 | 0.570589 |
| M.LDL.P | shift work: < 2 years | -0.06865 | 0.061183 | 0.263254 |
| M.LDL.L | shift work: < 2 years | -0.06705 | 0.061512 | 0.277006 |
| M.LDL.PL | shift work: < 2 years | -0.03714 | 0.044117 | 0.400879 |
| M.LDL.C | shift work: < 2 years | -0.08533 | 0.073054 | 0.244216 |
| M.LDL.CE | shift work: < 2 years | -0.11665 | 0.093214 | 0.212232 |
| M.LDL.FC | shift work: < 2 years | -0.05 | 0.044522 | 0.262738 |
| M.LDL.TG | shift work: < 2 years | -0.04798 | 0.059484 | 0.420938 |
| S.LDL.P | shift work: < 2 years | -0.06197 | 0.056334 | 0.272642 |
| S.LDL.L | shift work: < 2 years | -0.05549 | 0.056237 | 0.324999 |
| S.LDL.PL | shift work: < 2 years | -0.02389 | 0.038156 | 0.53204 |
| S.LDL.C | shift work: < 2 years | -0.1024 | 0.07413 | 0.16866 |
| S.LDL.CE | shift work: < 2 years | -0.10637 | 0.090211 | 0.239696 |
| S.LDL.FC | shift work: < 2 years | -0.05153 | 0.043885 | 0.241717 |
| S.LDL.TG | shift work: < 2 years | -0.0264 | 0.061337 | 0.667411 |
| XL.HDL.P | shift work: < 2 years | -0.01323 | 0.087947 | 0.880614 |
| XL.HDL.L | shift work: < 2 years | 0.012987 | 0.089128 | 0.884303 |
| XL.HDL.PL | shift work: < 2 years | -0.06176 | 0.102088 | 0.545912 |
| XL.HDL.C | shift work: < 2 years | 0.062891 | 0.089689 | 0.484038 |
| XL.HDL.CE | shift work: < 2 years | 0.045359 | 0.087572 | 0.6051 |
| XL.HDL.FC | shift work: < 2 years | 0.052461 | 0.09647 | 0.587216 |
| XL.HDL.TG | shift work: < 2 years | -0.0035 | 0.098654 | 0.971715 |
| L.HDL.P | shift work: < 2 years | -0.13008 | 0.081033 | 0.110078 |
| L.HDL.L | shift work: < 2 years | -0.14536 | 0.084275 | 0.086174 |
| L.HDL.PL | shift work: < 2 years | -0.13365 | 0.073968 | 0.072336 |
| L.HDL.C | shift work: < 2 years | -0.19667 | 0.099346 | 0.049136 |
| L.HDL.CE | shift work: < 2 years | -0.14846 | 0.093607 | 0.114393 |
| L.HDL.FC | shift work: < 2 years | -0.2238 | 0.122953 | 0.070228 |
| L.HDL.TG | shift work: < 2 years | -0.26279 | 0.128517 | 0.042194 |
| M.HDL.P | shift work: < 2 years | -0.0473 | 0.038394 | 0.219476 |
| M.HDL.L | shift work: < 2 years | -0.0329 | 0.03873 | 0.396608 |
| M.HDL.PL | shift work: < 2 years | -0.02273 | 0.038424 | 0.554855 |
| M.HDL.C | shift work: < 2 years | -0.05907 | 0.041231 | 0.153504 |
| M.HDL.CE | shift work: < 2 years | -0.03899 | 0.040127 | 0.332362 |
| M.HDL.FC | shift work: < 2 years | -0.07003 | 0.054583 | 0.200975 |
| M.HDL.TG | shift work: < 2 years | -0.00428 | 0.074656 | 0.954352 |
| S.HDL.P | shift work: < 2 years | -0.02403 | 0.025587 | 0.348761 |
| S.HDL.L | shift work: < 2 years | -0.01766 | 0.025795 | 0.494378 |
| S.HDL.PL | shift work: < 2 years | 0.006706 | 0.029162 | 0.818373 |
| S.HDL.C | shift work: < 2 years | -0.06824 | 0.036736 | 0.064672 |
| S.HDL.CE | shift work: < 2 years | -0.10507 | 0.050033 | 0.036928 |
| S.HDL.FC | shift work: < 2 years | -0.02285 | 0.028979 | 0.431408 |
| S.HDL.TG | shift work: < 2 years | 0.00313 | 0.058724 | 0.957549 |
| XXL.VLDL.PL_. | shift work: < 2 years | 0.059155 | 0.093792 | 0.528945 |
| XXL.VLDL.C_. | shift work: < 2 years | -0.0463 | 0.184675 | 0.802307 |
| XXL.VLDL.CE_. | shift work: < 2 years | -0.16221 | 0.325945 | 0.61926 |
| XXL.VLDL.FC_. | shift work: < 2 years | 0.027615 | 0.21607 | 0.898435 |
| XXL.VLDL.TG_. | shift work: < 2 years | 0.058124 | 0.063438 | 0.360624 |
| XL.VLDL.PL_. | shift work: < 2 years | -0.15775 | 0.353029 | 0.655465 |
| XL.VLDL.C_. | shift work: < 2 years | -0.13622 | 0.121858 | 0.265159 |
| XL.VLDL.CE_. | shift work: < 2 years | -0.34755 | 0.441482 | 0.432126 |
| XL.VLDL.FC_. | shift work: < 2 years | -0.1442 | 0.386443 | 0.70947 |
| XL.VLDL.TG_. | shift work: < 2 years | 0.015002 | 0.136628 | 0.912682 |
| L.VLDL.PL_. | shift work: < 2 years | -0.04683 | 0.037066 | 0.20793 |
| L.VLDL.C_. | shift work: < 2 years | -0.06328 | 0.062903 | 0.315717 |
| L.VLDL.CE_. | shift work: < 2 years | -0.15812 | 0.085487 | 0.065824 |
| L.VLDL.FC_. | shift work: < 2 years | 0.034297 | 0.226375 | 0.879733 |
| L.VLDL.TG_. | shift work: < 2 years | -0.01705 | 0.027704 | 0.539108 |
| M.VLDL.PL_. | shift work: < 2 years | -0.00936 | 0.01044 | 0.37093 |
| M.VLDL.C_. | shift work: < 2 years | 0.054758 | 0.055406 | 0.324329 |
| M.VLDL.CE_. | shift work: < 2 years | 0.000155 | 0.06211 | 0.998008 |
| M.VLDL.FC_. | shift work: < 2 years | 0.071219 | 0.054314 | 0.191336 |
| M.VLDL.TG_. | shift work: < 2 years | 0.005922 | 0.022136 | 0.789348 |
| S.VLDL.PL_. | shift work: < 2 years | 0.000587 | 0.015625 | 0.970083 |
| S.VLDL.C_. | shift work: < 2 years | -0.06391 | 0.036669 | 0.082898 |
| S.VLDL.CE_. | shift work: < 2 years | -0.05183 | 0.065045 | 0.426441 |
| S.VLDL.FC_. | shift work: < 2 years | -0.0229 | 0.014701 | 0.120959 |
| S.VLDL.TG_. | shift work: < 2 years | 0.052297 | 0.028856 | 0.071453 |
| XS.VLDL.PL_. | shift work: < 2 years | -0.03304 | 0.01857 | 0.076698 |
| XS.VLDL.C_. | shift work: < 2 years | -0.00286 | 0.014629 | 0.845162 |
| XS.VLDL.CE_. | shift work: < 2 years | 0.015482 | 0.022175 | 0.486004 |
| XS.VLDL.FC_. | shift work: < 2 years | -0.04677 | 0.020245 | 0.021842 |
| XS.VLDL.TG_. | shift work: < 2 years | 0.042511 | 0.041919 | 0.311775 |
| IDL.PL_. | shift work: < 2 years | -0.00081 | 0.006292 | 0.897279 |
| IDL.C_. | shift work: < 2 years | -0.00243 | 0.008145 | 0.765677 |
| IDL.CE_. | shift work: < 2 years | 0.006644 | 0.011592 | 0.567144 |
| IDL.FC_. | shift work: < 2 years | -0.03803 | 0.016618 | 0.023108 |
| IDL.TG_. | shift work: < 2 years | 0.040298 | 0.044292 | 0.364035 |
| L.LDL.PL_. | shift work: < 2 years | 0.016698 | 0.01177 | 0.157598 |
| L.LDL.C_. | shift work: < 2 years | -0.00884 | 0.009382 | 0.347341 |
| L.LDL.CE_. | shift work: < 2 years | -0.01103 | 0.016121 | 0.49474 |
| L.LDL.FC_. | shift work: < 2 years | -0.00751 | 0.011314 | 0.507695 |
| L.LDL.TG_. | shift work: < 2 years | 0.035967 | 0.046846 | 0.443542 |
| M.LDL.PL_. | shift work: < 2 years | 0.030137 | 0.021927 | 0.170868 |
| M.LDL.C_. | shift work: < 2 years | -0.02129 | 0.015597 | 0.173765 |
| M.LDL.CE_. | shift work: < 2 years | -0.04874 | 0.036797 | 0.18675 |
| M.LDL.FC_. | shift work: < 2 years | 0.030763 | 0.019958 | 0.124768 |
| M.LDL.TG_. | shift work: < 2 years | 0.035177 | 0.049861 | 0.48134 |
| S.LDL.PL_. | shift work: < 2 years | 0.039724 | 0.023241 | 0.088973 |
| S.LDL.C_. | shift work: < 2 years | -0.02479 | 0.018814 | 0.189128 |
| S.LDL.CE_. | shift work: < 2 years | -0.05926 | 0.038904 | 0.129155 |
| S.LDL.FC_. | shift work: < 2 years | 0.034907 | 0.017078 | 0.042248 |
| S.LDL.TG_. | shift work: < 2 years | 0.007243 | 0.048863 | 0.88231 |
| XL.HDL.PL_. | shift work: < 2 years | -0.07938 | 0.031642 | 0.012874 |
| XL.HDL.C_. | shift work: < 2 years | 0.04596 | 0.021025 | 0.030031 |
| XL.HDL.CE_. | shift work: < 2 years | 0.061321 | 0.02754 | 0.027074 |
| XL.HDL.FC_. | shift work: < 2 years | 0.029962 | 0.015365 | 0.052703 |
| XL.HDL.TG_. | shift work: < 2 years | 0.048251 | 0.086004 | 0.575396 |
| L.HDL.PL_. | shift work: < 2 years | 0.003602 | 0.013862 | 0.795244 |
| L.HDL.C_. | shift work: < 2 years | -0.01823 | 0.0176 | 0.301615 |
| L.HDL.CE_. | shift work: < 2 years | -0.00838 | 0.014166 | 0.554864 |
| L.HDL.FC_. | shift work: < 2 years | -0.06639 | 0.060728 | 0.275613 |
| L.HDL.TG_. | shift work: < 2 years | -0.01827 | 0.096513 | 0.850058 |
| M.HDL.PL_. | shift work: < 2 years | 0.007687 | 0.005533 | 0.166258 |
| M.HDL.C_. | shift work: < 2 years | -0.01303 | 0.008877 | 0.143691 |
| M.HDL.CE_. | shift work: < 2 years | -0.00786 | 0.010572 | 0.458148 |
| M.HDL.FC_. | shift work: < 2 years | -0.04921 | 0.019984 | 0.01464 |
| M.HDL.TG_. | shift work: < 2 years | 0.029308 | 0.066383 | 0.659343 |
| S.HDL.PL_. | shift work: < 2 years | 0.023134 | 0.013937 | 0.09853 |
| S.HDL.C_. | shift work: < 2 years | -0.05679 | 0.023521 | 0.016668 |
| S.HDL.CE_. | shift work: < 2 years | -0.08906 | 0.037899 | 0.019742 |
| S.HDL.FC_. | shift work: < 2 years | -0.00169 | 0.013653 | 0.901515 |
| S.HDL.TG_. | shift work: < 2 years | 0.037544 | 0.04956 | 0.449596 |
| VLDL.D | shift work: < 2 years | 0.006005 | 0.008116 | 0.46028 |
| LDL.D | shift work: < 2 years | 0.001567 | 0.000822 | 0.057915 |
| HDL.D | shift work: < 2 years | -0.00807 | 0.005679 | 0.156903 |
| Serum.C | shift work: < 2 years | -0.05237 | 0.042768 | 0.222247 |
| VLDL.C | shift work: < 2 years | -0.03622 | 0.086548 | 0.676081 |
| Remnant.C | shift work: < 2 years | -0.04068 | 0.065051 | 0.532459 |
| LDL.C | shift work: < 2 years | -0.09204 | 0.067828 | 0.176346 |
| HDL.C | shift work: < 2 years | -0.06224 | 0.044377 | 0.162329 |
| HDL2.C | shift work: < 2 years | -0.07778 | 0.062134 | 0.212136 |
| HDL3.C | shift work: < 2 years | -0.01827 | 0.013833 | 0.188108 |
| EstC | shift work: < 2 years | -0.0541 | 0.043757 | 0.21783 |
| FreeC | shift work: < 2 years | -0.03756 | 0.04146 | 0.366077 |
| Serum.TG | shift work: < 2 years | 0.022091 | 0.08231 | 0.788689 |
| VLDL.TG | shift work: < 2 years | 0.014351 | 0.120584 | 0.905388 |
| LDL.TG | shift work: < 2 years | -0.02376 | 0.058794 | 0.686603 |
| HDL.TG | shift work: < 2 years | 0.018798 | 0.054806 | 0.731991 |
| TotPG | shift work: < 2 years | -0.01539 | 0.037646 | 0.683171 |
| TG.PG | shift work: < 2 years | -0.00051 | 0.082523 | 0.995092 |
| PC | shift work: < 2 years | -0.0134 | 0.037056 | 0.718053 |
| SM | shift work: < 2 years | -0.0346 | 0.034939 | 0.323294 |
| TotCho | shift work: < 2 years | -0.01578 | 0.031903 | 0.621512 |
| ApoA1 | shift work: < 2 years | -0.02943 | 0.026065 | 0.260221 |
| ApoB | shift work: < 2 years | -0.00961 | 0.046272 | 0.835648 |
| ApoB.ApoA1 | shift work: < 2 years | -0.00868 | 0.047362 | 0.854721 |
| TotFA | shift work: < 2 years | -0.02415 | 0.041994 | 0.565843 |
| UnSat | shift work: < 2 years | -0.02442 | 0.010307 | 0.018794 |
| DHA | shift work: < 2 years | -0.14835 | 0.088676 | 0.095894 |
| LA | shift work: < 2 years | -0.0634 | 0.04244 | 0.136875 |
| FAw3 | shift work: < 2 years | -0.09544 | 0.066043 | 0.14998 |
| FAw6 | shift work: < 2 years | -0.07203 | 0.038378 | 0.062041 |
| PUFA | shift work: < 2 years | -0.06619 | 0.039119 | 0.092239 |
| MUFA | shift work: < 2 years | 0.024537 | 0.052862 | 0.643053 |
| SFA | shift work: < 2 years | -0.00718 | 0.045728 | 0.875313 |
| DHA.FA | shift work: < 2 years | -0.09295 | 0.069061 | 0.179844 |
| LA.FA | shift work: < 2 years | -0.02292 | 0.023488 | 0.33037 |
| FAw3.FA | shift work: < 2 years | -0.05061 | 0.043333 | 0.244196 |
| FAw6.FA | shift work: < 2 years | -0.03342 | 0.01731 | 0.054937 |
| PUFA.FA | shift work: < 2 years | -0.02261 | 0.017158 | 0.18901 |
| MUFA.FA | shift work: < 2 years | 0.015614 | 0.018588 | 0.401961 |
| SFA.FA | shift work: < 2 years | 0.007369 | 0.009239 | 0.426052 |
| Glc | shift work: < 2 years | 0.036017 | 0.032677 | 0.271825 |
| Lac | shift work: < 2 years | -0.09496 | 0.053875 | 0.0796 |
| Cit | shift work: < 2 years | 0.032876 | 0.032683 | 0.315801 |
| Ala | shift work: < 2 years | -0.01284 | 0.033339 | 0.70063 |
| Gln | shift work: < 2 years | 0.011348 | 0.034989 | 0.746074 |
| His | shift work: < 2 years | 0.027646 | 0.032684 | 0.39871 |
| Ile | shift work: < 2 years | 0.052146 | 0.071812 | 0.468644 |
| Leu | shift work: < 2 years | -0.04015 | 0.069449 | 0.563901 |
| Val | shift work: < 2 years | 0.082231 | 0.055499 | 0.14012 |
| Phe | shift work: < 2 years | 0.030485 | 0.040574 | 0.453386 |
| Tyr | shift work: < 2 years | 0.024293 | 0.063023 | 0.70031 |
| Ace | shift work: < 2 years | 0.007786 | 0.053877 | 0.885247 |
| AcAce | shift work: < 2 years | 0.079184 | 0.094284 | 0.40201 |
| bOHBut | shift work: < 2 years | 0.020116 | 0.070577 | 0.775949 |
| Crea | shift work: < 2 years | 0.012052 | 0.029502 | 0.683365 |
| Alb | shift work: < 2 years | -0.01374 | 0.011236 | 0.222882 |
| Gp | shift work: < 2 years | 0.022831 | 0.033736 | 0.499361 |
| XXL.VLDL.P | shift work: >5 years | -0.05361 | 0.160525 | 0.738722 |
| XXL.VLDL.L | shift work: >5 years | 0.022963 | 0.164298 | 0.888974 |
| XXL.VLDL.PL | shift work: >5 years | -0.08018 | 0.19443 | 0.680461 |
| XXL.VLDL.C | shift work: >5 years | -0.09241 | 0.255655 | 0.718119 |
| XXL.VLDL.CE | shift work: >5 years | -0.10846 | 0.330486 | 0.743082 |
| XXL.VLDL.FC | shift work: >5 years | -0.07568 | 0.246018 | 0.758692 |
| XXL.VLDL.TG | shift work: >5 years | -0.02689 | 0.153855 | 0.861423 |
| XL.VLDL.P | shift work: >5 years | -0.15705 | 0.382791 | 0.682006 |
| XL.VLDL.L | shift work: >5 years | -0.0051 | 0.375532 | 0.989173 |
| XL.VLDL.PL | shift work: >5 years | -0.2376 | 0.607505 | 0.696109 |
| XL.VLDL.C | shift work: >5 years | -0.12684 | 0.448469 | 0.777593 |
| XL.VLDL.CE | shift work: >5 years | -0.31547 | 0.523696 | 0.547538 |
| XL.VLDL.FC | shift work: >5 years | -0.24907 | 0.451206 | 0.581521 |
| XL.VLDL.TG | shift work: >5 years | 0.063397 | 0.370476 | 0.864284 |
| L.VLDL.P | shift work: >5 years | -0.08853 | 0.160095 | 0.580862 |
| L.VLDL.L | shift work: >5 years | 0.059231 | 0.164549 | 0.719232 |
| L.VLDL.PL | shift work: >5 years | 0.005469 | 0.15625 | 0.972111 |
| L.VLDL.C | shift work: >5 years | -0.02949 | 0.165332 | 0.858622 |
| L.VLDL.CE | shift work: >5 years | -0.18501 | 0.161897 | 0.254462 |
| L.VLDL.FC | shift work: >5 years | -0.11169 | 0.301108 | 0.711056 |
| L.VLDL.TG | shift work: >5 years | 0.037691 | 0.160792 | 0.814894 |
| M.VLDL.P | shift work: >5 years | -0.0473 | 0.087207 | 0.588103 |
| M.VLDL.L | shift work: >5 years | 0.008581 | 0.090127 | 0.924238 |
| M.VLDL.PL | shift work: >5 years | -0.00456 | 0.084392 | 0.956974 |
| M.VLDL.C | shift work: >5 years | -0.02146 | 0.103511 | 0.835962 |
| M.VLDL.CE | shift work: >5 years | 0.017316 | 0.096117 | 0.857204 |
| M.VLDL.FC | shift work: >5 years | -0.05834 | 0.125496 | 0.642482 |
| M.VLDL.TG | shift work: >5 years | -0.04132 | 0.091408 | 0.651676 |
| S.VLDL.P | shift work: >5 years | -0.04322 | 0.059638 | 0.469425 |
| S.VLDL.L | shift work: >5 years | -0.03393 | 0.059733 | 0.57064 |
| S.VLDL.PL | shift work: >5 years | -0.03766 | 0.052587 | 0.474782 |
| S.VLDL.C | shift work: >5 years | -0.08406 | 0.069904 | 0.230541 |
| S.VLDL.CE | shift work: >5 years | 0.009884 | 0.093255 | 0.915684 |
| S.VLDL.FC | shift work: >5 years | -0.06205 | 0.063413 | 0.328936 |
| S.VLDL.TG | shift work: >5 years | -0.03076 | 0.064822 | 0.635593 |
| XS.VLDL.P | shift work: >5 years | -0.01247 | 0.038914 | 0.748971 |
| XS.VLDL.L | shift work: >5 years | -0.01618 | 0.038777 | 0.676851 |
| XS.VLDL.PL | shift work: >5 years | -0.01613 | 0.041777 | 0.699911 |
| XS.VLDL.C | shift work: >5 years | -0.00658 | 0.041848 | 0.875158 |
| XS.VLDL.CE | shift work: >5 years | -0.003 | 0.042791 | 0.94412 |
| XS.VLDL.FC | shift work: >5 years | -0.02178 | 0.04499 | 0.628864 |
| XS.VLDL.TG | shift work: >5 years | 0.007725 | 0.046927 | 0.869406 |
| IDL.P | shift work: >5 years | -0.00236 | 0.037575 | 0.949924 |
| IDL.L | shift work: >5 years | 0.00198 | 0.037825 | 0.95831 |
| IDL.PL | shift work: >5 years | 0.001797 | 0.035149 | 0.959276 |
| IDL.C | shift work: >5 years | -0.01075 | 0.041275 | 0.794814 |
| IDL.CE | shift work: >5 years | -0.00276 | 0.042205 | 0.94796 |
| IDL.FC | shift work: >5 years | -0.00374 | 0.042232 | 0.9295 |
| IDL.TG | shift work: >5 years | 0.030699 | 0.039963 | 0.443287 |
| L.LDL.P | shift work: >5 years | -0.01346 | 0.040173 | 0.737958 |
| L.LDL.L | shift work: >5 years | -0.01445 | 0.040186 | 0.719507 |
| L.LDL.PL | shift work: >5 years | -0.00788 | 0.032288 | 0.807454 |
| L.LDL.C | shift work: >5 years | -0.01715 | 0.045283 | 0.70529 |
| L.LDL.CE | shift work: >5 years | -0.03061 | 0.049746 | 0.538983 |
| L.LDL.FC | shift work: >5 years | -0.01214 | 0.037331 | 0.745305 |
| L.LDL.TG | shift work: >5 years | 0.025116 | 0.041884 | 0.549423 |
| M.LDL.P | shift work: >5 years | -0.0248 | 0.042839 | 0.563312 |
| M.LDL.L | shift work: >5 years | -0.01053 | 0.04317 | 0.807555 |
| M.LDL.PL | shift work: >5 years | -0.00422 | 0.030958 | 0.891721 |
| M.LDL.C | shift work: >5 years | -0.02377 | 0.051336 | 0.643854 |
| M.LDL.CE | shift work: >5 years | -0.04753 | 0.065563 | 0.469262 |
| M.LDL.FC | shift work: >5 years | 5.46E-05 | 0.03128 | 0.998609 |
| M.LDL.TG | shift work: >5 years | 0.014286 | 0.041713 | 0.732359 |
| S.LDL.P | shift work: >5 years | -0.02932 | 0.039443 | 0.458122 |
| S.LDL.L | shift work: >5 years | -0.01851 | 0.039535 | 0.640194 |
| S.LDL.PL | shift work: >5 years | -0.00999 | 0.026889 | 0.710538 |
| S.LDL.C | shift work: >5 years | -0.01952 | 0.052509 | 0.710476 |
| S.LDL.CE | shift work: >5 years | -0.0259 | 0.063722 | 0.68479 |
| S.LDL.FC | shift work: >5 years | -0.01849 | 0.030867 | 0.549901 |
| S.LDL.TG | shift work: >5 years | -0.00777 | 0.043128 | 0.857121 |
| XL.HDL.P | shift work: >5 years | -0.01848 | 0.06129 | 0.763277 |
| XL.HDL.L | shift work: >5 years | -0.01341 | 0.062336 | 0.829845 |
| XL.HDL.PL | shift work: >5 years | -0.02883 | 0.071628 | 0.687692 |
| XL.HDL.C | shift work: >5 years | 0.006968 | 0.062906 | 0.911906 |
| XL.HDL.CE | shift work: >5 years | -0.00594 | 0.061551 | 0.923187 |
| XL.HDL.FC | shift work: >5 years | -0.01102 | 0.067663 | 0.870764 |
| XL.HDL.TG | shift work: >5 years | -0.02084 | 0.069614 | 0.764943 |
| L.HDL.P | shift work: >5 years | -0.07255 | 0.056701 | 0.202182 |
| L.HDL.L | shift work: >5 years | -0.07372 | 0.058906 | 0.212243 |
| L.HDL.PL | shift work: >5 years | -0.06633 | 0.051948 | 0.203133 |
| L.HDL.C | shift work: >5 years | -0.08021 | 0.069679 | 0.25098 |
| L.HDL.CE | shift work: >5 years | -0.07764 | 0.065368 | 0.236343 |
| L.HDL.FC | shift work: >5 years | -0.08976 | 0.086351 | 0.299801 |
| L.HDL.TG | shift work: >5 years | 0.00467 | 0.090274 | 0.958789 |
| M.HDL.P | shift work: >5 years | -0.04438 | 0.027121 | 0.10328 |
| M.HDL.L | shift work: >5 years | -0.03651 | 0.027379 | 0.183845 |
| M.HDL.PL | shift work: >5 years | -0.03208 | 0.027121 | 0.238251 |
| M.HDL.C | shift work: >5 years | -0.06194 | 0.029214 | 0.035152 |
| M.HDL.CE | shift work: >5 years | -0.046 | 0.028523 | 0.108282 |
| M.HDL.FC | shift work: >5 years | -0.05217 | 0.038522 | 0.177059 |
| M.HDL.TG | shift work: >5 years | -0.01429 | 0.052393 | 0.785318 |
| S.HDL.P | shift work: >5 years | -0.02032 | 0.018018 | 0.260675 |
| S.HDL.L | shift work: >5 years | -0.01661 | 0.018174 | 0.361877 |
| S.HDL.PL | shift work: >5 years | -0.0202 | 0.020554 | 0.326794 |
| S.HDL.C | shift work: >5 years | -0.01094 | 0.026234 | 0.677038 |
| S.HDL.CE | shift work: >5 years | -0.02025 | 0.035728 | 0.57137 |
| S.HDL.FC | shift work: >5 years | -0.02333 | 0.020532 | 0.257153 |
| S.HDL.TG | shift work: >5 years | 0.035277 | 0.041823 | 0.399908 |
| XXL.VLDL.PL_. | shift work: >5 years | -0.01485 | 0.067155 | 0.825219 |
| XXL.VLDL.C_. | shift work: >5 years | -0.00074 | 0.132655 | 0.995548 |
| XXL.VLDL.CE_. | shift work: >5 years | -0.04538 | 0.235089 | 0.847117 |
| XXL.VLDL.FC_. | shift work: >5 years | -0.04683 | 0.154609 | 0.762266 |
| XXL.VLDL.TG_. | shift work: >5 years | -0.00236 | 0.045713 | 0.958911 |
| XL.VLDL.PL_. | shift work: >5 years | -0.10726 | 0.254062 | 0.673299 |
| XL.VLDL.C_. | shift work: >5 years | -0.1097 | 0.087823 | 0.213101 |
| XL.VLDL.CE_. | shift work: >5 years | -0.3124 | 0.319268 | 0.328949 |
| XL.VLDL.FC_. | shift work: >5 years | -0.20766 | 0.278752 | 0.457146 |
| XL.VLDL.TG_. | shift work: >5 years | 0.011372 | 0.099097 | 0.908744 |
| L.VLDL.PL_. | shift work: >5 years | -0.0117 | 0.02654 | 0.659658 |
| L.VLDL.C_. | shift work: >5 years | -0.04078 | 0.045122 | 0.367165 |
| L.VLDL.CE_. | shift work: >5 years | -0.07646 | 0.061621 | 0.215959 |
| L.VLDL.FC_. | shift work: >5 years | -0.03499 | 0.162214 | 0.829412 |
| L.VLDL.TG_. | shift work: >5 years | -0.01863 | 0.019922 | 0.350706 |
| M.VLDL.PL_. | shift work: >5 years | -0.00834 | 0.007445 | 0.264059 |
| M.VLDL.C_. | shift work: >5 years | 0.052519 | 0.039419 | 0.184287 |
| M.VLDL.CE_. | shift work: >5 years | 0.044584 | 0.044291 | 0.315265 |
| M.VLDL.FC_. | shift work: >5 years | 0.009662 | 0.039205 | 0.805563 |
| M.VLDL.TG_. | shift work: >5 years | -0.01264 | 0.015747 | 0.422895 |
| S.VLDL.PL_. | shift work: >5 years | -0.00707 | 0.011128 | 0.526104 |
| S.VLDL.C_. | shift work: >5 years | -0.02624 | 0.025912 | 0.312396 |
| S.VLDL.CE_. | shift work: >5 years | -0.01669 | 0.04607 | 0.717427 |
| S.VLDL.FC_. | shift work: >5 years | -0.01726 | 0.010445 | 0.100038 |
| S.VLDL.TG_. | shift work: >5 years | 0.022965 | 0.020518 | 0.2643 |
| XS.VLDL.PL_. | shift work: >5 years | -0.01244 | 0.013145 | 0.344872 |
| XS.VLDL.C_. | shift work: >5 years | 0.003429 | 0.010563 | 0.745825 |
| XS.VLDL.CE_. | shift work: >5 years | 0.018109 | 0.015929 | 0.25702 |
| XS.VLDL.FC_. | shift work: >5 years | -0.02267 | 0.014448 | 0.117946 |
| XS.VLDL.TG_. | shift work: >5 years | 0.008803 | 0.029808 | 0.768031 |
| IDL.PL_. | shift work: >5 years | 0.001426 | 0.004451 | 0.748927 |
| IDL.C_. | shift work: >5 years | -0.0037 | 0.005827 | 0.525633 |
| IDL.CE_. | shift work: >5 years | -0.00058 | 0.008291 | 0.944263 |
| IDL.FC_. | shift work: >5 years | -0.00705 | 0.011834 | 0.552176 |
| IDL.TG_. | shift work: >5 years | 0.021644 | 0.031573 | 0.493756 |
| L.LDL.PL_. | shift work: >5 years | 0.005283 | 0.008245 | 0.522388 |
| L.LDL.C_. | shift work: >5 years | -0.00617 | 0.006655 | 0.354936 |
| L.LDL.CE_. | shift work: >5 years | -0.01285 | 0.011407 | 0.261041 |
| L.LDL.FC_. | shift work: >5 years | 0.006063 | 0.007982 | 0.448319 |
| L.LDL.TG_. | shift work: >5 years | 0.03375 | 0.033304 | 0.31202 |
| M.LDL.PL_. | shift work: >5 years | 0.012205 | 0.015451 | 0.430453 |
| M.LDL.C_. | shift work: >5 years | -0.01046 | 0.011051 | 0.34495 |
| M.LDL.CE_. | shift work: >5 years | -0.02223 | 0.026061 | 0.394623 |
| M.LDL.FC_. | shift work: >5 years | 0.008308 | 0.014081 | 0.555775 |
| M.LDL.TG_. | shift work: >5 years | 0.03181 | 0.035368 | 0.369453 |
| S.LDL.PL_. | shift work: >5 years | 0.010512 | 0.016367 | 0.5214 |
| S.LDL.C_. | shift work: >5 years | -0.00429 | 0.013325 | 0.748035 |
| S.LDL.CE_. | shift work: >5 years | -0.01526 | 0.027587 | 0.580676 |
| S.LDL.FC_. | shift work: >5 years | 0.009537 | 0.012047 | 0.429411 |
| S.LDL.TG_. | shift work: >5 years | 0.004285 | 0.034706 | 0.901863 |
| XL.HDL.PL_. | shift work: >5 years | -0.01855 | 0.022455 | 0.409714 |
| XL.HDL.C_. | shift work: >5 years | 0.013241 | 0.014917 | 0.375763 |
| XL.HDL.CE_. | shift work: >5 years | 0.025859 | 0.019729 | 0.191339 |
| XL.HDL.FC_. | shift work: >5 years | 0.007201 | 0.010929 | 0.510718 |
| XL.HDL.TG_. | shift work: >5 years | 0.00557 | 0.061373 | 0.927765 |
| L.HDL.PL_. | shift work: >5 years | 0.006933 | 0.009742 | 0.477453 |
| L.HDL.C_. | shift work: >5 years | -0.00858 | 0.012311 | 0.486543 |
| L.HDL.CE_. | shift work: >5 years | -0.00166 | 0.009934 | 0.867653 |
| L.HDL.FC_. | shift work: >5 years | -0.00602 | 0.04284 | 0.888363 |
| L.HDL.TG_. | shift work: >5 years | 0.117055 | 0.067777 | 0.085628 |
| M.HDL.PL_. | shift work: >5 years | 0.010731 | 0.003958 | 0.007229 |
| M.HDL.C_. | shift work: >5 years | -0.01334 | 0.006313 | 0.035745 |
| M.HDL.CE_. | shift work: >5 years | -0.01309 | 0.007524 | 0.083474 |
| M.HDL.FC_. | shift work: >5 years | -0.01324 | 0.014038 | 0.346597 |
| M.HDL.TG_. | shift work: >5 years | 0.030147 | 0.046958 | 0.521573 |
| S.HDL.PL_. | shift work: >5 years | 0.004852 | 0.01004 | 0.629356 |
| S.HDL.C_. | shift work: >5 years | -0.01205 | 0.016681 | 0.470897 |
| S.HDL.CE_. | shift work: >5 years | -0.01181 | 0.026829 | 0.660287 |
| S.HDL.FC_. | shift work: >5 years | -0.0049 | 0.009711 | 0.61458 |
| S.HDL.TG_. | shift work: >5 years | 0.065967 | 0.035589 | 0.065124 |
| VLDL.D | shift work: >5 years | -0.00311 | 0.005737 | 0.588816 |
| LDL.D | shift work: >5 years | 0.001691 | 0.000592 | 0.004683 |
| HDL.D | shift work: >5 years | -0.00328 | 0.003968 | 0.409903 |
| Serum.C | shift work: >5 years | -0.02199 | 0.030013 | 0.464615 |
| VLDL.C | shift work: >5 years | -0.03159 | 0.060933 | 0.604659 |
| Remnant.C | shift work: >5 years | -0.02011 | 0.045657 | 0.660105 |
| LDL.C | shift work: >5 years | -0.02965 | 0.047584 | 0.533912 |
| HDL.C | shift work: >5 years | -0.04043 | 0.031199 | 0.19646 |
| HDL2.C | shift work: >5 years | -0.044 | 0.04355 | 0.313512 |
| HDL3.C | shift work: >5 years | -0.00814 | 0.009761 | 0.405119 |
| EstC | shift work: >5 years | -0.02917 | 0.030772 | 0.344195 |
| FreeC | shift work: >5 years | -0.00498 | 0.029066 | 0.864153 |
| Serum.TG | shift work: >5 years | -0.00922 | 0.057873 | 0.873524 |
| VLDL.TG | shift work: >5 years | 0.000438 | 0.085443 | 0.995911 |
| LDL.TG | shift work: >5 years | 0.013925 | 0.0411 | 0.735111 |
| HDL.TG | shift work: >5 years | 0.024844 | 0.038786 | 0.522539 |
| TotPG | shift work: >5 years | -0.02378 | 0.026594 | 0.372247 |
| TG.PG | shift work: >5 years | -0.0678 | 0.058326 | 0.246419 |
| PC | shift work: >5 years | -0.02348 | 0.026198 | 0.371247 |
| SM | shift work: >5 years | -0.00836 | 0.024672 | 0.734972 |
| TotCho | shift work: >5 years | -0.01748 | 0.022484 | 0.437865 |
| ApoA1 | shift work: >5 years | -0.02647 | 0.018438 | 0.152656 |
| ApoB | shift work: >5 years | -0.00243 | 0.03246 | 0.940368 |
| ApoB.ApoA1 | shift work: >5 years | -0.00221 | 0.033029 | 0.946812 |
| TotFA | shift work: >5 years | -0.02743 | 0.029527 | 0.354085 |
| UnSat | shift work: >5 years | -0.01313 | 0.007313 | 0.074033 |
| DHA | shift work: >5 years | -0.11821 | 0.06283 | 0.061264 |
| LA | shift work: >5 years | -0.04569 | 0.029829 | 0.127181 |
| FAw3 | shift work: >5 years | -0.08108 | 0.046562 | 0.083064 |
| FAw6 | shift work: >5 years | -0.04352 | 0.026998 | 0.108534 |
| PUFA | shift work: >5 years | -0.04421 | 0.027493 | 0.10936 |
| MUFA | shift work: >5 years | -0.00704 | 0.037295 | 0.850513 |
| SFA | shift work: >5 years | -0.00937 | 0.032326 | 0.772252 |
| DHA.FA | shift work: >5 years | -0.09073 | 0.049309 | 0.06715 |
| LA.FA | shift work: >5 years | 0.001234 | 0.016588 | 0.940788 |
| FAw3.FA | shift work: >5 years | -0.06438 | 0.030728 | 0.037335 |
| FAw6.FA | shift work: >5 years | -0.00662 | 0.012225 | 0.588815 |
| PUFA.FA | shift work: >5 years | -0.00718 | 0.012102 | 0.553445 |
| MUFA.FA | shift work: >5 years | 0.002058 | 0.013175 | 0.876045 |
| SFA.FA | shift work: >5 years | 0.008865 | 0.006585 | 0.179708 |
| Glc | shift work: >5 years | -0.00779 | 0.023668 | 0.74224 |
| Lac | shift work: >5 years | -0.10803 | 0.038914 | 0.005997 |
| Cit | shift work: >5 years | 0.047859 | 0.023577 | 0.04366 |
| Ala | shift work: >5 years | -0.04009 | 0.02407 | 0.097314 |
| Gln | shift work: >5 years | 0.014314 | 0.025126 | 0.569537 |
| His | shift work: >5 years | 0.019014 | 0.023549 | 0.420327 |
| Ile | shift work: >5 years | 0.00632 | 0.05189 | 0.903183 |
| Leu | shift work: >5 years | -0.06372 | 0.049651 | 0.200831 |
| Val | shift work: >5 years | 0.025991 | 0.039833 | 0.514814 |
| Phe | shift work: >5 years | 0.039989 | 0.028996 | 0.169355 |
| Tyr | shift work: >5 years | 0.00963 | 0.04524 | 0.831627 |
| Ace | shift work: >5 years | -0.01839 | 0.038651 | 0.634693 |
| AcAce | shift work: >5 years | 0.033506 | 0.068219 | 0.623808 |
| bOHBut | shift work: >5 years | 0.020426 | 0.051365 | 0.691278 |
| Crea | shift work: >5 years | 0.027764 | 0.020904 | 0.185616 |
| Alb | shift work: >5 years | -0.00319 | 0.008094 | 0.69365 |
| Gp | shift work: >5 years | -0.00389 | 0.024097 | 0.872018 |

**Visual display of univariate analyses (n=69). Acute effects measured directly after a night-shift**

**Fig 1** Univariate mixed-effect linear regression models (confidence intervals). Acute effects of the night-shift directly before blood sampling compared with blood sampling during a day session among night-shift workers (n = 69, 184 samples). 1 SD increment in biomarker concentration. A = minimal confounder model, associations are adjusted for age. B = confounder model, associations are adjusted for age, and BMI. C = full-covariate model, associations are adjusted for age, BMI, chronotype, blood sampling time, saturated fat intake, and timing of last meal before blood draw. Abbreviations: TotCho = Total Cholesterol, C = Cholesterol, VLDL = very low density lipoprotein, LDL = low density lipoprotein, IDL = intermediate density lipoprotein, HDL = high-density lipoprotein (HDL2 particle density 1.063-1.125 g/mL, HDL3 1.125-1.210 g/mL), XL = extra-large, L= large, M = medium, S= small, TG = triglycerides, TotPG = Total phosphoglycerides, TotFA = Total fatty acids, FAw3 = Omega-3 fatty acids, FAw6 = Omega-6 fatty acids, PUFA = poly-unsaturated fatty acids, MUFA = mono-unsaturated fatty acids, SFA = saturated fatty acids, LA = linoleic acid, DHA = docosahexaenoic acid, LA.FA = Ratio of 18:2 linoleic acid to total fatty acids, DHA.FA = Ratio of 22:6 docosahexaenoic acid to total fatty acids, Ala = alanine, Gln = glutamine, His = histidine, Ile = isoleucine, Leu = leucine, Val = valine, Phe = phenylalanine, Tyr = tyrosine, Glc = glucose, Lac = Lactate, Cit = citrate, Ace = acetate, AcAce = acetoacetate, bOHBut = β-hydroxybutyrate, ApoA1 = apolipoprotein A-I, ApoB = apolipoprotein B, ApoB.ApoA1 = ratio of apolipoprotein B to apolipoprotein A-I

| **Supplementary table III. Estimates and standard errors for metabolite associations of acute effects of nightshift work (measured directly after a night shift compared with blood sampling during a day session). Univariate linear regression models.** | | | | |
| --- | --- | --- | --- | --- |
| Metabolite | Trait | Estimates | Standard deviations | p-values |
| XXL.VLDL.P | night shift | -0.18828 | 0.223773 | 0.401284 |
| XXL.VLDL.L | night shift | -0.22357 | 0.233244 | 0.339112 |
| XXL.VLDL.PL | night shift | -0.2777 | 0.261172 | 0.289184 |
| XXL.VLDL.C | night shift | -0.31726 | 0.36225 | 0.382351 |
| XXL.VLDL.CE | night shift | -0.66288 | 0.492136 | 0.179702 |
| XXL.VLDL.FC | night shift | -0.10523 | 0.331207 | 0.751079 |
| XXL.VLDL.TG | night shift | -0.14904 | 0.204075 | 0.466198 |
| XL.VLDL.P | night shift | -0.24099 | 0.569168 | 0.672515 |
| XL.VLDL.L | night shift | -0.31238 | 0.549365 | 0.570357 |
| XL.VLDL.PL | night shift | -0.51122 | 0.929417 | 0.582984 |
| XL.VLDL.C | night shift | -0.45828 | 0.670161 | 0.494981 |
| XL.VLDL.CE | night shift | -0.33098 | 0.807727 | 0.682463 |
| XL.VLDL.FC | night shift | -0.28805 | 0.691218 | 0.677382 |
| XL.VLDL.TG | night shift | -0.29489 | 0.554602 | 0.595575 |
| L.VLDL.P | night shift | 0.147162 | 0.198827 | 0.460337 |
| L.VLDL.L | night shift | 0.01056 | 0.226513 | 0.96287 |
| L.VLDL.PL | night shift | 0.046357 | 0.200393 | 0.817352 |
| L.VLDL.C | night shift | 0.057663 | 0.219225 | 0.79286 |
| L.VLDL.CE | night shift | 0.044739 | 0.211111 | 0.832446 |
| L.VLDL.FC | night shift | 0.084185 | 0.439031 | 0.848164 |
| L.VLDL.TG | night shift | 0.167279 | 0.208713 | 0.424048 |
| M.VLDL.P | night shift | -0.0646 | 0.09576 | 0.500976 |
| M.VLDL.L | night shift | -0.06703 | 0.100185 | 0.504477 |
| M.VLDL.PL | night shift | -0.07955 | 0.104285 | 0.446734 |
| M.VLDL.C | night shift | -0.09182 | 0.14442 | 0.525739 |
| M.VLDL.CE | night shift | -0.03157 | 0.111923 | 0.778281 |
| M.VLDL.FC | night shift | -0.17114 | 0.171846 | 0.320693 |
| M.VLDL.TG | night shift | -0.0787 | 0.108619 | 0.469848 |
| S.VLDL.P | night shift | -0.04753 | 0.055904 | 0.396779 |
| S.VLDL.L | night shift | -0.06588 | 0.063901 | 0.304262 |
| S.VLDL.PL | night shift | -0.02668 | 0.047951 | 0.57891 |
| S.VLDL.C | night shift | -0.06763 | 0.0707 | 0.340429 |
| S.VLDL.CE | night shift | -0.09989 | 0.100284 | 0.320725 |
| S.VLDL.FC | night shift | -0.05184 | 0.063847 | 0.418252 |
| S.VLDL.TG | night shift | -0.05286 | 0.066907 | 0.430785 |
| XS.VLDL.P | night shift | 0.003173 | 0.03103 | 0.918733 |
| XS.VLDL.L | night shift | 0.003199 | 0.032524 | 0.921802 |
| XS.VLDL.PL | night shift | 0.000604 | 0.035491 | 0.986456 |
| XS.VLDL.C | night shift | 0.011271 | 0.041577 | 0.786713 |
| XS.VLDL.CE | night shift | 0.028783 | 0.045383 | 0.526949 |
| XS.VLDL.FC | night shift | -0.03315 | 0.047234 | 0.483952 |
| XS.VLDL.TG | night shift | 0.000463 | 0.043346 | 0.991486 |
| IDL.P | night shift | 0.013047 | 0.033551 | 0.697999 |
| IDL.L | night shift | 0.012102 | 0.034943 | 0.729633 |
| IDL.PL | night shift | 0.008387 | 0.032546 | 0.79702 |
| IDL.C | night shift | 0.010136 | 0.042033 | 0.8098 |
| IDL.CE | night shift | 0.015555 | 0.043932 | 0.723811 |
| IDL.FC | night shift | -0.0028 | 0.042814 | 0.947956 |
| IDL.TG | night shift | 0.04825 | 0.035431 | 0.175599 |
| L.LDL.P | night shift | -0.00914 | 0.03659 | 0.803063 |
| L.LDL.L | night shift | -0.01259 | 0.037754 | 0.739241 |
| L.LDL.PL | night shift | -0.00475 | 0.030789 | 0.877682 |
| L.LDL.C | night shift | -0.02268 | 0.045165 | 0.616339 |
| L.LDL.CE | night shift | -0.02251 | 0.049942 | 0.652944 |
| L.LDL.FC | night shift | -0.01235 | 0.037224 | 0.740567 |
| L.LDL.TG | night shift | 0.060179 | 0.036624 | 0.102831 |
| M.LDL.P | night shift | -0.0365 | 0.038907 | 0.349911 |
| M.LDL.L | night shift | -0.04052 | 0.040871 | 0.323253 |
| M.LDL.PL | night shift | -0.00483 | 0.030937 | 0.876035 |
| M.LDL.C | night shift | -0.06194 | 0.051407 | 0.230218 |
| M.LDL.CE | night shift | -0.07955 | 0.069092 | 0.251458 |
| M.LDL.FC | night shift | -0.026 | 0.03254 | 0.425567 |
| M.LDL.TG | night shift | 0.045047 | 0.038266 | 0.241299 |
| S.LDL.P | night shift | -0.03759 | 0.035647 | 0.293567 |
| S.LDL.L | night shift | -0.04039 | 0.036732 | 0.273417 |
| S.LDL.PL | night shift | -0.00309 | 0.027463 | 0.910688 |
| S.LDL.C | night shift | -0.07274 | 0.060149 | 0.228375 |
| S.LDL.CE | night shift | -0.08788 | 0.066747 | 0.189987 |
| S.LDL.FC | night shift | -0.03291 | 0.032814 | 0.317622 |
| S.LDL.TG | night shift | 0.019838 | 0.042184 | 0.638944 |
| XL.HDL.P | night shift | 0.127622 | 0.046413 | 0.006895 |
| XL.HDL.L | night shift | 0.132805 | 0.051965 | 0.011786 |
| XL.HDL.PL | night shift | 0.195973 | 0.066554 | 0.003817 |
| XL.HDL.C | night shift | 0.086961 | 0.060138 | 0.150502 |
| XL.HDL.CE | night shift | 0.062108 | 0.060658 | 0.307708 |
| XL.HDL.FC | night shift | 0.177417 | 0.061239 | 0.004409 |
| XL.HDL.TG | night shift | 0.101873 | 0.081688 | 0.214392 |
| L.HDL.P | night shift | 0.16015 | 0.052654 | 0.002861 |
| L.HDL.L | night shift | 0.15397 | 0.050945 | 0.003057 |
| L.HDL.PL | night shift | 0.135467 | 0.048995 | 0.006535 |
| L.HDL.C | night shift | 0.172007 | 0.06103 | 0.00561 |
| L.HDL.CE | night shift | 0.177339 | 0.054285 | 0.001417 |
| L.HDL.FC | night shift | 0.171366 | 0.092722 | 0.066736 |
| L.HDL.TG | night shift | 0.171577 | 0.094552 | 0.071675 |
| M.HDL.P | night shift | 0.060682 | 0.03177 | 0.058109 |
| M.HDL.L | night shift | 0.061575 | 0.03315 | 0.065231 |
| M.HDL.PL | night shift | 0.06885 | 0.0323 | 0.034707 |
| M.HDL.C | night shift | 0.053359 | 0.037733 | 0.159352 |
| M.HDL.CE | night shift | 0.048363 | 0.036873 | 0.191563 |
| M.HDL.FC | night shift | 0.07079 | 0.045618 | 0.122906 |
| M.HDL.TG | night shift | 0.092544 | 0.052364 | 0.079344 |
| S.HDL.P | night shift | 0.003779 | 0.019058 | 0.843098 |
| S.HDL.L | night shift | 0.003272 | 0.019583 | 0.867539 |
| S.HDL.PL | night shift | 0.021492 | 0.024022 | 0.372415 |
| S.HDL.C | night shift | -0.04636 | 0.03214 | 0.151127 |
| S.HDL.CE | night shift | -0.06549 | 0.042009 | 0.121066 |
| S.HDL.FC | night shift | 0.01515 | 0.023365 | 0.517733 |
| S.HDL.TG | night shift | 0.00211 | 0.053907 | 0.968829 |
| XXL.VLDL.PL_. | night shift | -0.14746 | 0.090814 | 0.106275 |
| XXL.VLDL.C_. | night shift | -0.25991 | 0.195535 | 0.185429 |
| XXL.VLDL.CE_. | night shift | -0.67618 | 0.360025 | 0.061921 |
| XXL.VLDL.FC_. | night shift | -0.17304 | 0.209864 | 0.410789 |
| XXL.VLDL.TG_. | night shift | -0.15065 | 0.067867 | 0.027652 |
| XL.VLDL.PL_. | night shift | -0.34543 | 0.400558 | 0.389617 |
| XL.VLDL.C_. | night shift | -0.09322 | 0.137029 | 0.4972 |
| XL.VLDL.CE_. | night shift | -0.14364 | 0.525951 | 0.785073 |
| XL.VLDL.FC_. | night shift | -0.175 | 0.448699 | 0.696984 |
| XL.VLDL.TG_. | night shift | -0.17463 | 0.162198 | 0.282983 |
| L.VLDL.PL_. | night shift | -0.00554 | 0.039881 | 0.889579 |
| L.VLDL.C_. | night shift | 0.008416 | 0.071069 | 0.90587 |
| L.VLDL.CE_. | night shift | -0.01193 | 0.099386 | 0.904563 |
| L.VLDL.FC_. | night shift | 0.07064 | 0.244889 | 0.773333 |
| L.VLDL.TG_. | night shift | -0.00053 | 0.030791 | 0.986199 |
| M.VLDL.PL_. | night shift | 0.010802 | 0.010034 | 0.283098 |
| M.VLDL.C_. | night shift | 0.035169 | 0.047695 | 0.461827 |
| M.VLDL.CE_. | night shift | 0.055671 | 0.061589 | 0.367248 |
| M.VLDL.FC_. | night shift | -0.0485 | 0.06104 | 0.427843 |
| M.VLDL.TG_. | night shift | -0.01583 | 0.022038 | 0.473539 |
| S.VLDL.PL_. | night shift | 0.024992 | 0.015064 | 0.098895 |
| S.VLDL.C_. | night shift | -0.01146 | 0.030896 | 0.711159 |
| S.VLDL.CE_. | night shift | -0.01514 | 0.058116 | 0.794724 |
| S.VLDL.FC_. | night shift | 0.009737 | 0.014118 | 0.491428 |
| S.VLDL.TG_. | night shift | -0.00277 | 0.026667 | 0.917449 |
| XS.VLDL.PL_. | night shift | -0.00664 | 0.016176 | 0.681935 |
| XS.VLDL.C_. | night shift | 0.016837 | 0.016546 | 0.310208 |
| XS.VLDL.CE_. | night shift | 0.036999 | 0.022803 | 0.106558 |
| XS.VLDL.FC_. | night shift | -0.04265 | 0.02119 | 0.045662 |
| XS.VLDL.TG_. | night shift | -0.01322 | 0.039255 | 0.736684 |
| IDL.PL_. | night shift | -0.00619 | 0.005242 | 0.239366 |
| IDL.C_. | night shift | -0.00194 | 0.009 | 0.829996 |
| IDL.CE_. | night shift | 0.002724 | 0.012847 | 0.832328 |
| IDL.FC_. | night shift | -0.01798 | 0.015941 | 0.26105 |
| IDL.TG_. | night shift | 0.040672 | 0.043128 | 0.346978 |
| L.LDL.PL_. | night shift | 0.007579 | 0.007418 | 0.308811 |
| L.LDL.C_. | night shift | -0.01041 | 0.008893 | 0.243398 |
| L.LDL.CE_. | night shift | -0.01383 | 0.014708 | 0.348304 |
| L.LDL.FC_. | night shift | -0.00073 | 0.00897 | 0.935486 |
| L.LDL.TG_. | night shift | 0.084903 | 0.045291 | 0.062608 |
| M.LDL.PL_. | night shift | 0.035127 | 0.017013 | 0.040635 |
| M.LDL.C_. | night shift | -0.0243 | 0.014342 | 0.091996 |
| M.LDL.CE_. | night shift | -0.04284 | 0.034092 | 0.21063 |
| M.LDL.FC_. | night shift | 0.021212 | 0.014841 | 0.155047 |
| M.LDL.TG_. | night shift | 0.098644 | 0.046336 | 0.034781 |
| S.LDL.PL_. | night shift | 0.040854 | 0.018223 | 0.02641 |
| S.LDL.C_. | night shift | -0.02956 | 0.017148 | 0.086573 |
| S.LDL.CE_. | night shift | -0.05243 | 0.037 | 0.158291 |
| S.LDL.FC_. | night shift | 0.01271 | 0.0139 | 0.361909 |
| S.LDL.TG_. | night shift | 0.068078 | 0.045472 | 0.13631 |
| XL.HDL.PL_. | night shift | 0.047494 | 0.030992 | 0.127403 |
| XL.HDL.C_. | night shift | -0.05395 | 0.018308 | 0.003702 |
| XL.HDL.CE_. | night shift | -0.08145 | 0.02632 | 0.002313 |
| XL.HDL.FC_. | night shift | 0.032635 | 0.013865 | 0.019719 |
| XL.HDL.TG_. | night shift | -0.05815 | 0.08471 | 0.493314 |
| L.HDL.PL_. | night shift | -0.02531 | 0.008404 | 0.003135 |
| L.HDL.C_. | night shift | 0.023821 | 0.011262 | 0.036369 |
| L.HDL.CE_. | night shift | 0.024863 | 0.009615 | 0.010819 |
| L.HDL.FC_. | night shift | 0.00325 | 0.04993 | 0.948187 |
| L.HDL.TG_. | night shift | 0.01578 | 0.065705 | 0.810537 |
| M.HDL.PL_. | night shift | 0.008141 | 0.005684 | 0.153867 |
| M.HDL.C_. | night shift | -0.00752 | 0.00845 | 0.374503 |
| M.HDL.CE_. | night shift | -0.00911 | 0.0096 | 0.344179 |
| M.HDL.FC_. | night shift | 0.00696 | 0.015302 | 0.649936 |
| M.HDL.TG_. | night shift | 0.013881 | 0.057865 | 0.810732 |
| S.HDL.PL_. | night shift | 0.017748 | 0.015309 | 0.247804 |
| S.HDL.C_. | night shift | -0.03915 | 0.020804 | 0.0617 |
| S.HDL.CE_. | night shift | -0.06106 | 0.032627 | 0.063222 |
| S.HDL.FC_. | night shift | 0.017243 | 0.013358 | 0.198654 |
| S.HDL.TG_. | night shift | 0.000454 | 0.051646 | 0.99299 |
| VLDL.D | night shift | -0.00168 | 0.006872 | 0.807698 |
| LDL.D | night shift | 0.003536 | 0.000869 | 6.81E-05 |
| HDL.D | night shift | 0.007416 | 0.002854 | 0.010541 |
| Serum.C | night shift | 0.007129 | 0.030295 | 0.814299 |
| VLDL.C | night shift | -0.02476 | 0.067207 | 0.713117 |
| Remnant.C | night shift | -0.00276 | 0.046807 | 0.953029 |
| LDL.C | night shift | -0.04452 | 0.047523 | 0.350417 |
| HDL.C | night shift | 0.068025 | 0.03143 | 0.032236 |
| HDL2.C | night shift | 0.10237 | 0.042432 | 0.017231 |
| HDL3.C | night shift | 0.001601 | 0.01038 | 0.877673 |
| EstC | night shift | -0.00038 | 0.032477 | 0.990571 |
| FreeC | night shift | 0.024922 | 0.0277 | 0.369891 |
| Serum.TG | night shift | 0.016622 | 0.060969 | 0.785531 |
| VLDL.TG | night shift | -0.05065 | 0.098299 | 0.607149 |
| LDL.TG | night shift | 0.055645 | 0.035846 | 0.123098 |
| HDL.TG | night shift | 0.099771 | 0.04426 | 0.025631 |
| TotPG | night shift | 0.039882 | 0.032583 | 0.222887 |
| TG.PG | night shift | -0.0379 | 0.065667 | 0.564669 |
| PC | night shift | 0.038408 | 0.032172 | 0.234411 |
| SM | night shift | 0.038309 | 0.025246 | 0.131403 |
| TotCho | night shift | 0.036522 | 0.025975 | 0.161861 |
| ApoA1 | night shift | 0.038621 | 0.021047 | 0.068592 |
| ApoB | night shift | -0.01445 | 0.029754 | 0.627912 |
| ApoB.ApoA1 | night shift | -0.04417 | 0.024917 | 0.078799 |
| TotFA | night shift | 0.023076 | 0.032129 | 0.473825 |
| UnSat | night shift | -0.02394 | 0.009028 | 0.008813 |
| DHA | night shift | -0.17715 | 0.080017 | 0.028263 |
| LA | night shift | 0.022736 | 0.03146 | 0.471095 |
| FAw3 | night shift | -0.11465 | 0.052096 | 0.029294 |
| FAw6 | night shift | 0.014342 | 0.029775 | 0.630778 |
| PUFA | night shift | 0.001394 | 0.029651 | 0.962575 |
| MUFA | night shift | 0.037376 | 0.038754 | 0.3365 |
| SFA | night shift | 0.031883 | 0.039263 | 0.418041 |
| DHA.FA | night shift | -0.18744 | 0.068225 | 0.006666 |
| LA.FA | night shift | -0.00451 | 0.017998 | 0.802642 |
| FAw3.FA | night shift | -0.13574 | 0.037402 | 0.000381 |
| FAw6.FA | night shift | -0.009 | 0.014552 | 0.537318 |
| PUFA.FA | night shift | -0.02107 | 0.014168 | 0.139048 |
| MUFA.FA | night shift | 0.01059 | 0.01601 | 0.509279 |
| SFA.FA | night shift | 0.011989 | 0.009175 | 0.193045 |
| Glc | night shift | 0.052676 | 0.039954 | 0.189053 |
| Lac | night shift | -0.1688 | 0.058755 | 0.004532 |
| Cit | night shift | 0.020091 | 0.037368 | 0.591441 |
| Ala | night shift | -0.14583 | 0.03522 | 5.24E-05 |
| Gln | night shift | -0.06145 | 0.035828 | 0.088231 |
| His | night shift | -0.04471 | 0.037093 | 0.229632 |
| Ile | night shift | 0.001909 | 0.077203 | 0.980304 |
| Leu | night shift | -0.03162 | 0.068759 | 0.646116 |
| Val | night shift | -0.03686 | 0.058284 | 0.527888 |
| Phe | night shift | 0.001522 | 0.03964 | 0.969421 |
| Tyr | night shift | -0.05922 | 0.071297 | 0.407279 |
| Ace | night shift | -0.04946 | 0.058323 | 0.397488 |
| AcAce | night shift | 0.188043 | 0.110859 | 0.091472 |
| bOHBut | night shift | 0.14767 | 0.08878 | 0.097777 |
| Crea | night shift | -0.02448 | 0.026002 | 0.347913 |
| Alb | night shift | 0.02544 | 0.012783 | 0.048017 |
| Gp | night shift | 0.007519 | 0.029905 | 0.801821 |

| **Supplementary table IV. FDR-adjusted q-values of 57 metabolites used in regression analysis for chronic and acute effects of shift work (* < 0.05)** | | | |
| --- | --- | --- | --- |
| Metabolite | Trait | Q-value |  |
| XXL.VLDL.P | shift work: < 2 years | 0.938271 |  |
| XL.VLDL.P | shift work: < 2 years | 0.94687 |  |
| L.VLDL.P | shift work: < 2 years | 0.94687 |  |
| M.VLDL.P | shift work: < 2 years | 0.94687 |  |
| S.VLDL.P | shift work: < 2 years | 0.938271 |  |
| XS.VLDL.P | shift work: < 2 years | 0.938271 |  |
| IDL.P | shift work: < 2 years | 0.933532 |  |
| L.LDL.P | shift work: < 2 years | 0.882251 |  |
| M.LDL.P | shift work: < 2 years | 0.777029 |  |
| S.LDL.P | shift work: < 2 years | 0.777029 |  |
| XL.HDL.P | shift work: < 2 years | 0.94687 |  |
| L.HDL.P | shift work: < 2 years | 0.743508 |  |
| M.HDL.P | shift work: < 2 years | 0.745181 |  |
| S.HDL.P | shift work: < 2 years | 0.882251 |  |
| ApoA1 | shift work: < 2 years | 0.777029 |  |
| ApoB | shift work: < 2 years | 0.94687 |  |
| ApoB.ApoA1 | shift work: < 2 years | 0.94687 |  |
| Serum.TG | shift work: < 2 years | 0.946427 |  |
| VLDL.TG | shift work: < 2 years | 0.94692 |  |
| LDL.TG | shift work: < 2 years | 0.938271 |  |
| HDL.TG | shift work: < 2 years | 0.938271 |  |
| Serum.C | shift work: < 2 years | 0.745181 |  |
| VLDL.C | shift work: < 2 years | 0.938271 |  |
| Remnant.C | shift work: < 2 years | 0.938271 |  |
| LDL.C | shift work: < 2 years | 0.743508 |  |
| HDL.C | shift work: < 2 years | 0.743508 |  |
| HDL2.C | shift work: < 2 years | 0.745181 |  |
| HDL3.C | shift work: < 2 years | 0.743508 |  |
| TotFA | shift work: < 2 years | 0.938271 |  |
| FAw3 | shift work: < 2 years | 0.743508 |  |
| FAw6 | shift work: < 2 years | 0.743508 |  |
| PUFA | shift work: < 2 years | 0.743508 |  |
| MUFA | shift work: < 2 years | 0.938271 |  |
| SFA | shift work: < 2 years | 0.94687 |  |
| DHA | shift work: < 2 years | 0.743508 |  |
| LA | shift work: < 2 years | 0.743508 |  |
| DHA.FA | shift work: < 2 years | 0.743508 |  |
| LA.FA | shift work: < 2 years | 0.875865 |  |
| FAw3.FA | shift work: < 2 years | 0.777029 |  |
| FAw6.FA | shift work: < 2 years | 0.743508 |  |
| PUFA.FA | shift work: < 2 years | 0.743508 |  |
| MUFA.FA | shift work: < 2 years | 0.923671 |  |
| SFA.FA | shift work: < 2 years | 0.933532 |  |
| Glc | shift work: < 2 years | 0.777029 |  |
| Lac | shift work: < 2 years | 0.743508 |  |
| Cit | shift work: < 2 years | 0.857174 |  |
| Ace | shift work: < 2 years | 0.94687 |  |
| AcAce | shift work: < 2 years | 0.923671 |  |
| bOHBut | shift work: < 2 years | 0.941044 |  |
| Ala | shift work: < 2 years | 0.938271 |  |
| Gln | shift work: < 2 years | 0.938271 |  |
| His | shift work: < 2 years | 0.923671 |  |
| Ile | shift work: < 2 years | 0.933532 |  |
| Leu | shift work: < 2 years | 0.938271 |  |
| Val | shift work: < 2 years | 0.743508 |  |
| Phe | shift work: < 2 years | 0.933532 |  |
| Tyr | shift work: < 2 years | 0.938271 |  |
| XXL.VLDL.P | shift work: >5 years | 0.938271 |  |
| XL.VLDL.P | shift work: >5 years | 0.938271 |  |
| L.VLDL.P | shift work: >5 years | 0.938271 |  |
| M.VLDL.P | shift work: >5 years | 0.938271 |  |
| S.VLDL.P | shift work: >5 years | 0.933532 |  |
| XS.VLDL.P | shift work: >5 years | 0.938271 |  |
| IDL.P | shift work: >5 years | 0.958331 |  |
| L.LDL.P | shift work: >5 years | 0.938271 |  |
| M.LDL.P | shift work: >5 years | 0.938271 |  |
| S.LDL.P | shift work: >5 years | 0.933532 |  |
| XL.HDL.P | shift work: >5 years | 0.941044 |  |
| L.HDL.P | shift work: >5 years | 0.743508 |  |
| M.HDL.P | shift work: >5 years | 0.743508 |  |
| S.HDL.P | shift work: >5 years | 0.777029 |  |
| ApoA1 | shift work: >5 years | 0.743508 |  |
| ApoB | shift work: >5 years | 0.958331 |  |
| ApoB.ApoA1 | shift work: >5 years | 0.958331 |  |
| Serum.TG | shift work: >5 years | 0.94687 |  |
| VLDL.TG | shift work: >5 years | 0.995911 |  |
| LDL.TG | shift work: >5 years | 0.938271 |  |
| HDL.TG | shift work: >5 years | 0.938271 |  |
| Serum.C | shift work: >5 years | 0.933532 |  |
| VLDL.C | shift work: >5 years | 0.938271 |  |
| Remnant.C | shift work: >5 years | 0.938271 |  |
| LDL.C | shift work: >5 years | 0.938271 |  |
| HDL.C | shift work: >5 years | 0.743508 |  |
| HDL2.C | shift work: >5 years | 0.857174 |  |
| HDL3.C | shift work: >5 years | 0.923671 |  |
| TotFA | shift work: >5 years | 0.882251 |  |
| FAw3 | shift work: >5 years | 0.743508 |  |
| FAw6 | shift work: >5 years | 0.743508 |  |
| PUFA | shift work: >5 years | 0.743508 |  |
| MUFA | shift work: >5 years | 0.94687 |  |
| SFA | shift work: >5 years | 0.941044 |  |
| DHA | shift work: >5 years | 0.743508 |  |
| LA | shift work: >5 years | 0.743508 |  |
| DHA.FA | shift work: >5 years | 0.743508 |  |
| LA.FA | shift work: >5 years | 0.958331 |  |
| FAw3.FA | shift work: >5 years | 0.743508 |  |
| FAw6.FA | shift work: >5 years | 0.938271 |  |
| PUFA.FA | shift work: >5 years | 0.938271 |  |
| MUFA.FA | shift work: >5 years | 0.94687 |  |
| SFA.FA | shift work: >5 years | 0.743508 |  |
| Glc | shift work: >5 years | 0.938271 |  |
| Lac | shift work: >5 years | 0.683659 |  |
| Cit | shift work: >5 years | 0.743508 |  |
| Ace | shift work: >5 years | 0.938271 |  |
| AcAce | shift work: >5 years | 0.938271 |  |
| bOHBut | shift work: >5 years | 0.938271 |  |
| Ala | shift work: >5 years | 0.743508 |  |
| Gln | shift work: >5 years | 0.938271 |  |
| His | shift work: >5 years | 0.933532 |  |
| Ile | shift work: >5 years | 0.94692 |  |
| Leu | shift work: >5 years | 0.743508 |  |
| Val | shift work: >5 years | 0.938271 |  |
| Phe | shift work: >5 years | 0.743508 |  |
| Tyr | shift work: >5 years | 0.94687 |  |
| XXL.VLDL.P | night shift | 0.768655 |  |
| XL.VLDL.P | night shift | 0.871213 |  |
| L.VLDL.P | night shift | 0.794354 |  |
| M.VLDL.P | night shift | 0.805977 |  |
| S.VLDL.P | night shift | 0.768655 |  |
| XS.VLDL.P | night shift | 0.980304 |  |
| IDL.P | night shift | 0.883645 |  |
| L.LDL.P | night shift | 0.928301 |  |
| M.LDL.P | night shift | 0.768221 |  |
| S.LDL.P | night shift | 0.727536 |  |
| XL.HDL.P | night shift | 0.065499 |  |
| L.HDL.P | night shift | 0.054366 |  |
| M.HDL.P | night shift | 0.276019 |  |
| S.HDL.P | night shift | 0.942286 |  |
| ApoA1 | night shift | 0.300751 |  |
| ApoB | night shift | 0.856056 |  |
| ApoB.ApoA1 | night shift | 0.320824 |  |
| Serum.TG | night shift | 0.928301 |  |
| VLDL.TG | night shift | 0.856056 |  |
| LDL.TG | night shift | 0.389811 |  |
| HDL.TG | night shift | 0.166976 |  |
| Serum.C | night shift | 0.928301 |  |
| VLDL.C | night shift | 0.883645 |  |
| Remnant.C | night shift | 0.980304 |  |
| LDL.C | night shift | 0.768221 |  |
| HDL.C | night shift | 0.167039 |  |
| HDL2.C | night shift | 0.140308 |  |
| HDL3.C | night shift | 0.962065 |  |
| TotFA | night shift | 0.794354 |  |
| FAw3 | night shift | 0.166976 |  |
| FAw6 | night shift | 0.856056 |  |
| PUFA | night shift | 0.980304 |  |
| MUFA | night shift | 0.768221 |  |
| SFA | night shift | 0.768655 |  |
| DHA | night shift | 0.166976 |  |
| LA | night shift | 0.794354 |  |
| DHA.FA | night shift | 0.065499 |  |
| LA.FA | night shift | 0.928301 |  |
| FAw3.FA | night shift | **0.010857*** |  |
| FAw6.FA | night shift | 0.805977 |  |
| PUFA.FA | night shift | 0.417143 |  |
| MUFA.FA | night shift | 0.805977 |  |
| SFA.FA | night shift | 0.523979 |  |
| Glc | night shift | 0.523979 |  |
| Lac | night shift | 0.064577 |  |
| Cit | night shift | 0.856056 |  |
| Ace | night shift | 0.768655 |  |
| AcAce | night shift | 0.325868 |  |
| bOHBut | night shift | 0.32784 |  |
| Ala | night shift | **0.002989*** |  |
| Gln | night shift | 0.325868 |  |
| His | night shift | 0.594955 |  |
| Ile | night shift | 0.980304 |  |
| Leu | night shift | 0.856479 |  |
| Val | night shift | 0.805977 |  |
| Phe | night shift | 0.980304 |  |
| Tyr | night shift | 0.768655 |  |
